# Synthesis of isoprenoid bisphosphonate ethers through C–P bond formations: Potential inhibitors of geranylgeranyl diphosphate synthase

**DOI:** 10.3762/bjoc.10.171

**Published:** 2014-07-18

**Authors:** Xiang Zhou, Jacqueline E Reilly, Kathleen A Loerch, Raymond J Hohl, David F Wiemer

**Affiliations:** 1 Department of Chemistry, University of Iowa, Iowa City, Iowa 52242-1294, USA; 2 Department of Pharmacology, University of Iowa, Iowa City, Iowa 52242-1294, USA; 3 Department of Internal Medicine, University of Iowa, Iowa City, Iowa 52242-1294, USA

**Keywords:** bisphosphonate, isoprenoid biosynthesis, organophosphorous, phosphonate formation

## Abstract

A set of bisphosphonate ethers has been prepared through sequential phosphonylation and alkylation of monophosphonate ethers. After formation of the corresponding phosphonic acid salts, these compounds were tested for their ability to inhibit the enzyme geranylgeranyl diphosphate synthase (GGDPS). Five of the new compounds show IC_50_ values of less than 1 μM against GGDPS with little to no activity against the related enzyme farnesyl diphosphate synthase (FDPS). The most active compound displayed an IC_50_ value of 82 nM when assayed with GGDPS, and no activity against FDPS even at a 10 μM concentration.

## Introduction

**Figure 1:**
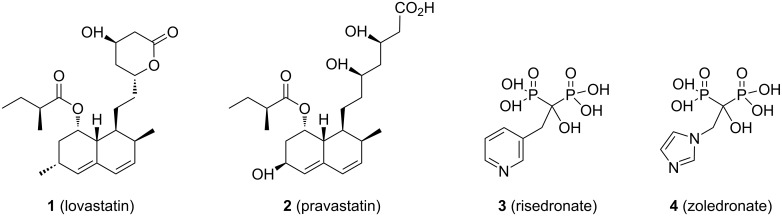
Inhibitors of isoprene biosynthesis.

Several enzymes of the isoprenoid biosynthesis pathways are the targets of widely prescribed drugs. For example, hydroxymethylglutaryl CoA reductase (HMGCoA) is viewed as the first committed step of isoprenoid and steroid biosynthesis, and is the target of the statin class of cholesterol-lowering agents including lovastatin (**1**, Figure 1) and pravastatin (**2**) [1]. The downstream enzyme farnesyl diphosphate synthase (FDPS) is the target of the nitrogenous bisphosphonates including risedronate (**3**) and zoledronate (**4**), which are widely used for treatment of osteoporosis [2]. It can be argued that the success of these drugs is due at least in part to the central roles that isoprenoids play in mammalian metabolism, which suggests that other enzymes in these pathways also may have value as drug targets.

**Figure 2:**
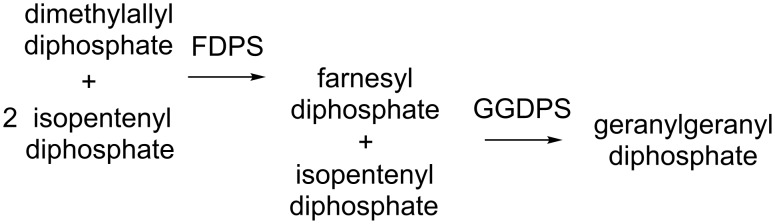
Biosynthesis of geranylgeranyl diphosphate.

One of our interests in isoprenoid biosynthesis has been the enzyme geranylgeranyl diphosphate synthase (GGDPS), which mediates the reaction of the C_15_ compound farnesyl diphosphate (FPP) with the C_5_ isopentenyl diphosphate to form the C_20_ isoprenoid geranylgeranyl diphosphate (GGPP) (Figure 2) [3]. Geranylgeranylation is an important posttranslational modification, especially among proteins in the Ras superfamily of small GTPases that are involved in a variety of signaling pathways [4]. Based on the premise that inhibition of GGDPS should reduce cellular levels of GGPP and thus diminish protein geranylgeranylation, one might expect that inhibitors of this enzyme would interfere with essential cell signaling pathways and demonstrate antiproliferative activity.

**Figure 3:**
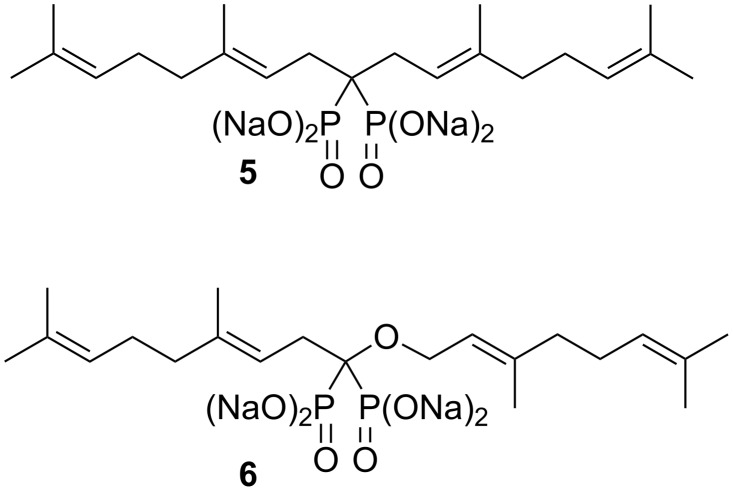
A known inhibitor of GGDPS (**5**) and a new analogue (**6**).

Several years ago we reported the synthesis of digeranyl bisphosphonate (DGBP, **5**, Figure 3) [5], and determined that this compound was an inhibitor of GGDPS (IC_50_ ~ 200 nM), competitive with FPP, and yet showed much less activity against FDPS (IC_50_ > 10 μM) in enzyme assays [6]. Furthermore, despite the high degree of negative charge on DGBP at physiological pH, Western blot analyses of K562 cells (a human-derived, myeloid leukemia cell line) treated with this compound make clear that it penetrates the cell membrane at a concentration sufficient to impact GGPP levels. For example in the presence of micromolar DGBP, Rap1a which is normally found to be fully geranylgeranylated through posttranslational processing, instead is only partially modified [5]. Preparation of a prodrug form of DGBP does increase the impact of the drug by nearly an order of magnitude [7], but masking the negative charges of DGBP is not essential for observation of cellular activity. Following our reports on the activity of DGBP, a beautiful set of crystallographic analyses from the Oldfield group attributed the activity of this compound and a number of others in part to a V-like shape [8]. This shape allows one geranyl group to occupy the enzyme channel where FPP enters the active site of GGDPS, while at the same time the second isoprenoid chain can fit nicely in the groove where the product GGPP normally departs from the active site.

To continue efforts [9] to increase the potency of GGDPS inhibitors, we sought a new set of isoprenoid bisphosphonates as represented by structure **6** (Figure 3). This *O*,*C*-digeranyl geminal bisphosphonate was expected to preserve a V-like structure very similar to that of DGBP. However, the presence of an oxygen substituent on the geminal carbon should lower the p*K*_a_ of bisphosphonate **6** relative to that of compound **5**, which might enhance its similarity to an isoprenoid diphosphate. In both monophosphonates [10] and bisphosphonates [11] introduction of an alpha hydroxy group has been reported to increase biological activity significantly. In bisphosphonates even a small change in p*K*_a3_ may be important because it lies in a range close to physiological pH [12]. If an ether substituent on this template had a comparable impact, it could significantly increase the activity relative to DGBP itself [13]. Furthermore, one binding model suggests that the hydroxy group itself, so prominent in the clinically used bisphosphonates, contributes only modestly to binding with the bone surface [14], and therefore might be a site appropriate for further modification. Thus we decided to pursue compounds of the general structure **6**. We report here the synthesis of some isoprenoid bisphosphonate ethers in this family and our initial studies of their biological activity.

## Results and Discussion

**Scheme 1:**
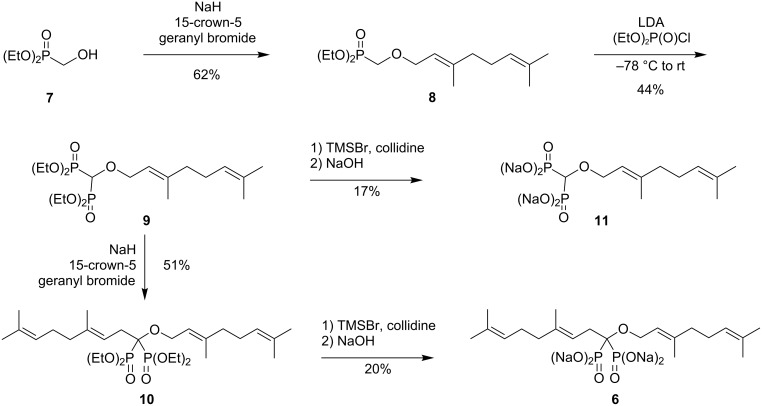
Synthesis of bisphosphonate ethers **6** and **11**.

Of the different routes one might consider to prepare geminal bisphosphonate ethers, some can be readily dismissed. For example, while several routes to hydroxybisphosphonates are known [15], any attempt to incorporate an ether linkage through the corresponding alkoxide after formation of the bisphosphonate would face the strong possibility of phosphonate–phosphate rearrangement [15–17]. However, diethyl hydroxymethylphosphonate (**7**, Scheme 1) is known to react with a base and geranyl bromide to afford the ether **8** in good yield [18]. With compound **8** in hand, formation of the second C–P bond occurred readily upon treatment with base and diethyl chlorophosphate [19–23] to give the bisphosphonate ether **9** in modest yield. Alkylation of ether **9** with geranyl bromide proceeded under conditions similar to those we have reported for the preparation of dialkyl bisphosphonate **5**, and gave the desired tetraethyl *O*,*C*-digeranylbisphosphonate **10**. Hydrolysis of the phosphonate esters proceeded under standard McKenna conditions [24], but only a limited amount of the product **6** was recovered after precipitation from acetone/water. A parallel hydrolysis of bisphosphonate **9** gave compound **11**, also in modest yield. Because the ^31^P NMR spectra of the reaction mixtures showed a single resonance in both cases, it is quite likely that the low yield results from low recovery of the bisphosphonate salts.

**Scheme 2:**
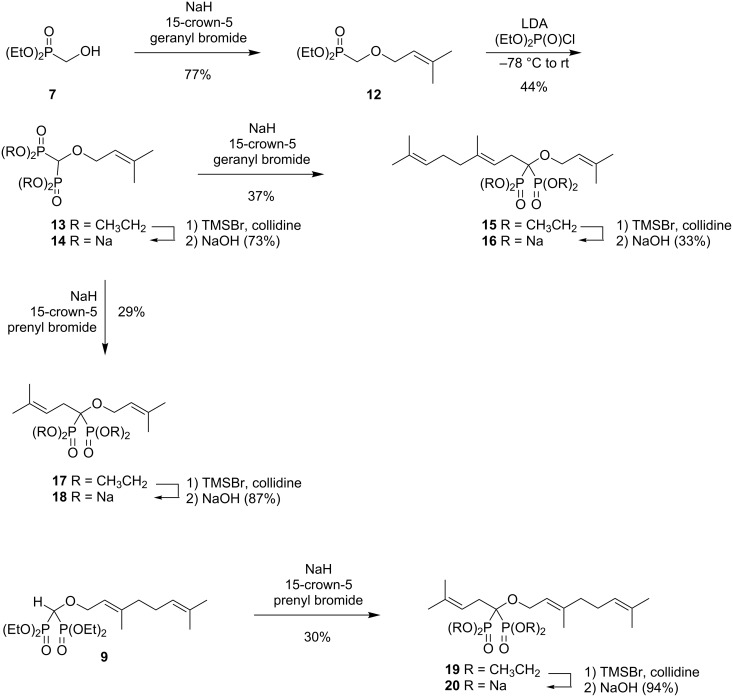
Synthesis of prenyl/geranyl bisphosphonate isomers.

Compound **6** should preserve the V-shape that would allow one isoprenoid chain to nestle within the FPP site while the other occupies the GGPP site [8]. It would not be readily apparent however, if one site is occupied preferentially by the *O*-geranyl group, or whether this group is randomly distributed between the two possibilities. In an initial effort to distinguish between random binding and differential binding, we have prepared the two isomeric bisphosphonate salts **16** and **20** through variations on the strategy used to prepare the digeranyl compound **6**. As shown in Scheme 2, reaction of phosphonate **7** with base and prenyl bromide gave the known phosphonate **12** [25]. Treatment of this phosphonate with base and diethyl chlorophosphate gave the desired bisphosphonate ester **13**. This ester was converted to the corresponding salt under standard conditions to obtain compound **14**. Alternatively, reaction of ester **13** with base and geranyl bromide gave the tetraethyl ester **15** and hydrolysis in this case afforded the desired phosphonate **16**. In a similar manner, reaction of the bisphosphonate ester **13** with base and prenyl bromide gave the *O,C*-diprenyl product **17**, and standard hydrolysis gave the salt **18**. To prepare the isomeric *O*-geranyl-*C*-prenyl compounds, the geranyl ether **9** was treated with base and prenyl bromide under parallel reaction conditions to afford compound **19**. Standard hydrolysis of this ester then gave the desired phosphonate salt **20**.

**Scheme 3:**
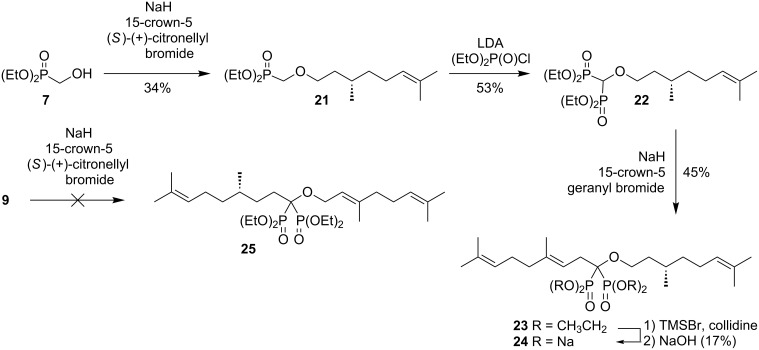
Synthesis of citronellyl bisphosphonates.

To gauge the generality of this approach to bisphosphonate ethers while still maintaining isoprenoid substructures, preparation of a citronellal series was examined. Alkylation of phosphonate **7** with (*S*)-(+)-citronellyl bromide occurred under the standard conditions, albeit in lower yield (Scheme 3). The resulting ether **21** was converted to the corresponding bisphosphonate **22** through formation of the anion and reaction with diethyl chlorophosphate. Alkylation of this bisphosphonate with geranyl bromide also proved feasible, and gave the expected tetraethyl ester **23**. Hydrolysis of compound **23** under standard conditions gave the desired salt **24**. In contrast, efforts to alkylate the *O*-geranyl bisphosphonate **9** with citronellyl bromide under parallel conditions went unrewarded, which might be attributed to the lower reactivity of this alkyl bromide vis-à-vis the allylic geranyl and prenyl bromides used above. Alternate strategies for preparation of compound **25** have not yet been explored, pending determination of the biological activity of the compounds in hand.

Preliminary evaluation of the biological activity of the dialkyl bisphosphonates was based on their ability to inhibit the enzymes GGDPS and FDPS [26]. The two prenyl bisphosphonate ethers, compounds **18** and **14**, showed little or no activity in these assays, as might be expected given their minimal isoprenoid chains [27]. However the compounds bearing longer alkyl chains were more interesting. As shown in Table 1, a range of activities was observed for these bisphosphonates. Under the specific conditions employed for the enzyme assays, compound **5** had an IC_50_ of 210 nM, which is very comparable to the value initially observed [6]. The *O*,*C*-digeranyl compound **6** was similar to this value which was disappointing, but the *O*-geranyl compound **11** could be considered surprisingly potent given the limited activity previously reported for geranyl bisphosphonate (10 μM) [27]. The two prenyl–geranyl isomers, compounds **16** and **20** differed by a factor of ~2.5 with one roughly as potent and one ~3 fold less potent than the digeranyl compound **5**. Our hypothesis was that random placement of the two isoprenoid chains should result in nearly identical biological activity for these isomeric compounds, while if placement of the isoprenoid chains were ordered then the two isomers might well show different biological activity. The observed difference is intriguing and may support the concept of an ordered binding. However, the most interesting result was observed with the citronellyl derivative **24**. This compound displayed an IC_50_ of 82 nM, which is ~2.6 fold more potent than the DGBP control (**5**). Furthermore, compound **24** displayed no activity in assays with FDPS, suggesting that its inhibition is highly selective.

**Table 1:** Activity of bisphosphonate ethers as inhibitors of GGDPS and FDPS.

Compound	GGDPS IC_50_ (nM)	FDPS IC_50_ (nM)

**5**	210	>10,000
**6**	408	>10,000
**11**	238	>10,000
**16**	684	830
**18**	4,750	5260
**20**	274	>10,000
**24**	82	>10,000
**4** (zoledronate)	ND	18

## Conclusion

In conclusion, we have prepared a family of bisphosphonate ethers that incorporated terpenoid elements designed to enhance their ability to inhibit the enzyme GGDPS. The increased potency observed with the citronellyl ether **24** versus compounds prepared earlier, as well as the difference in activity between the two prenyl–geranyl isomers, encourage a more extensive investigation of the biological activity of these compounds [28]. Such studies are ongoing and will be reported in due course.

## Supporting Information

File 1 Experimental procedures, characterization data, and ^1^H and ^13^C NMR spectra are provided for all new compounds.
