# Supplementary material for: Synthesis of isoprenoid bisphosphonate ethers through C–P bond formations: Potential inhibitors of geranylgeranyl diphosphate synthase
Source: Beilstein J Org Chem. 2014 Jul 18;10:1645–50. doi: 10.3762/bjoc.10.171 (PMC4142842; doi:10.3762/bjoc.10.171)
Supplement: File 1 — Experimental procedures, characterization data, and 1H and 13C NMR spectra are provided for all new compounds. [file Beilstein_J_Org_Chem-10-1645-s001.pdf]

Supporting information

for

Synthesis of isoprenoid bisphosphonate ethers through C–P bond formations: Potential inhibitors of geranylgeranyl diphosphate synthase

Xiang Zhou[1], Jacqueline E. Reilly[2], Kathleen A. Loerch[1], Raymond J. Hohl[2,3], and David F. Wiemer*[1,2,§]

Address: [1]Department of Chemistry, University of Iowa, Iowa City, Iowa 52242-1294, USA, [2]Department of Pharmacology, University of Iowa, Iowa City, Iowa 52242-1294, USA and [3]Department of Internal Medicine, University of Iowa, Iowa City, Iowa 52242-1294, USA

Email: David F. Wiemer - david-wiemer@uiowa.edu

*Corresponding author

Experimental procedures, characterization data, and ^{1}H and ^{13}C NMR spectra are provided for all new compounds

Supplemental Table of Contents

General experimental procedures	S4
Representative procedure for monophosphonate ether formation (**8**)	S4
Bisphosphonate ether **9**	S5
Representative procedure for bisphosphonate ether alkylation (**10**)	S5
Bisphosphonate salt **11**	S6
Bisphosphonate salt **6**	S6
Monophosphonate ether **12**	S6
Representative procedure for monophosphonate ether phosphonylation (**13**)	S7
Bisphosphonate salt **14**	S7
Bisphosphonate ester **15**	S8
Bisphosphonate salt **16**	S8
Bisphosphonate ester **17**	S8
Bisphosphonate salt **18**	S9
Bisphosphonate ester **19**	S9
Representative procedure for hydrolysis of bisphosphonate esters (**20**)	S9
Synthesis of monophosphonate ether **21**	S10
Synthesis of bisphosphonate ether **22**	S10
Synthesis of tetraethyl bisphosphonate ether **23**	S11
Synthesis of bisphosphonate salt **24**	S11
Procedure for the enzyme assays	S12
References	S12
^{1}H NMR for compound **8** (300 MHz)	S13
^{13}C NMR for compound **8** (75 MHz)	S14
^{1}H NMR for compound **9** (300 MHz)	S15
^{13}C NMR for compound **9** (75 MHz)	S16
^{1}H NMR for compound **10** (300 MHz)	S17
^{13}C NMR for compound **10** (75 MHz)	S18
^{1}H NMR for compound **11** (300 MHz)	S19
^{13}C NMR for compound **11** (75 MHz)	S20
^{1}H NMR for compound **6** (300 MHz)	S21
^{13}C NMR for compound **6** (125 MHz)	S22
^{1}H NMR for compound **12** (300 MHz)	S23
^{13}C NMR for compound **12** (75 MHz)	S24
^{1}H NMR for compound **13** (300 MHz)	S25
^{13}C NMR for compound **13** (75 MHz)	S26
^{1}H NMR for compound **14** (300 MHz)	S27
^{13}C NMR for compound **14** (100 MHz)	S28
^{1}H NMR for compound **15** (400 MHz)	S29
^{13}C NMR for compound **15** (100 MHz)	S30
^{1}H NMR for compound **16** (500 MHz)	S31
^{13}C NMR for compound **16** (125 MHz)	S32
^{1}H NMR for compound **17** (500 MHz)	S33
^{13}C NMR for compound **17** (125 MHz)	S34
^{1}H NMR for compound **18** (500 MHz)	S35

^{13}C NMR for compound **18** (125 MHz) S36
^{1}H NMR for compound **19** (300 MHz) S37
^{13}C NMR for compound **19** (75 MHz) S38
^{1}H NMR for compound **20** (500 MHz) S39
^{13}C NMR for compound **20** (125 MHz) S40
^{1}H NMR for compound **21** (500 MHz) S41
^{13}C NMR for compound **21** (125 MHz) S42
^{1}H NMR for compound **22** (400 MHz) S43
^{13}C NMR for compound **22** (100 MHz) S44
^{1}H NMR for compound **23** (400 MHz) S45
^{13}C NMR for compound **23** (100 MHz) S46
^{1}H NMR for compound **24** (500 MHz) S47
^{13}C NMR for compound **24** (125 MHz) S48

General experimental procedures. Tetrahydrofuran was freshly distilled from sodium/benzophenone, while methylene chloride was distilled from calcium hydride prior to use. All other reagents and solvents were purchased from commercial sources and used without further purification. All reactions in nonaqueous solvents were conducted in flame-dried glassware under a positive pressure of argon and with magnetic stirring. The NMR spectra were obtained at 300, 400, or 500 MHz for ^{1}H, and 75, 100, or 125 MHz for ^{13}C, with internal standards of $(CH_3)_4Si$ (^{1}H, 0.00) or $CDCl_3$ (^{1}H, 7.27; ^{13}C, 77.2 ppm) for non-aqueous samples or D_2O (^{1}H, 4.80) and 1,4-dioxane (^{13}C, 66.7 ppm) for aqueous samples. The ^{31}P chemical shifts were reported in ppm relative to 85% H_3PO_4 (external standard). High resolution mass spectra were obtained at the University of Iowa Mass Spectrometry Facility. Silica gel (60 Å, 0.040–0.063 mm) was used for flash chromatography.

Monophosphonate ether 8. Diethyl hydroxymethylphosphonate (**7**, 1 mL, 6.8 mmol) was added dropwise to a solution of NaH (60% dispersion in mineral oil, 300 mg, 7.5 mmol) in THF (7 mL) in an ice bath, followed by addition of 15-crown-5 (0.1 mL, 1 M in THF). After 30 minutes, geranyl bromide (1.62 g, 7.5 mmol) was added to the reaction mixture and it was allowed to react at room temperature overnight. Once the reaction was complete based on analysis of the ^{31}P NMR spectrum, saturated NH_4Cl was added. The resulting residue was extracted with Et_2O, the organic extracts were combined, dried (Na_2SO_4), concentrated in vacuo, and purified by column chromatography (5% EtOH in hexane) to afford the desired product **8** as a colorless oil (1.27 g, 62%): ^{1}H NMR (300 MHz, $CDCl_3$) δ 5.31 (t, J = 6.5 Hz, 1H), 5.08 (t, J = 4.9 Hz, 1H), 4.25–4.09 (m, 6H), 3.74 (d, J_{HP} = 8.6 Hz, 2H), 2.17–1.98 (m, 4H), 1.68 (s, 6H) 1.60

(s, 3H), 1.35 (t, J = 7.3 Hz, 6H); ^{13}C NMR (75 MHz, $CDCl_3$) δ 141.7, 131.5, 123.6, 119.6, 69.0 (d, J_{CP} = 12.7 Hz), 62.9 (d, J_{CP} = 166.0 Hz), 62.1 (d, J_{CP} = 6.1 Hz, 2C), 39.4, 26.0, 25.5, 17.4, 16.3, 16.2 (2C); ^{31}P NMR (121 MHz, $CDCl_3$) δ 22.0.

Bisphosphonate ether 9. Prepared according to the general procedure given for compound **13**: yield, 44%; colorless oil; ^{1}H NMR (300 MHz, $CDCl_3$) δ 5.26 (t, J = 7.0 Hz, 1H), 5.01 (t, J = 6.7 Hz, 1H), 4.27 (t, J = 7.5 Hz, 2H), 4.23–4.10 (m, 8H), 3.95 (t, J_{HP} = 17.6 Hz, 1H), 2.09–1.93 (m, 4H), 1.63 (s, 3H), 1.63 (s, 3H), 1.60 (s, 3H), 1.28 (t, J = 7.3 Hz, 12H); ^{13}C NMR (75 MHz, $CDCl_3$) δ 142.1, 131.0, 123.2, 119.0, 70.1 (t, J_{CP} = 157.9 Hz), 69.2 (t, J_{CP} =5.1 Hz), 62.7 (t, J_{CP} = 4.1 Hz, 2C), 62.5 (t, J_{CP} = 3.2 Hz, 2C), 39.1, 25.7, 25.0, 17.0, 15.9 (t, J_{CP} = 2.4 Hz, 2C), 15.8 (t, J_{CP} = 2.7 Hz, 2C), 15.8; ^{31}P NMR (121 MHz, $CDCl_3$) δ 16.1; HRMS (ES^+, *m*/*z*) calcd for $(M+Na)^+$ $C_{19}H_{38}O_7P_2Na$: 463.1991; found: 463.1972.

Bisphosphonate ether 10. Compound **9** (325 mg, 0.74 mmol) was added into a solution of NaH (60% dispersion in mineral oil, 50 mg, 1.25 mmol) in THF (3 mL), 15-crown-5 (0.1 mL, 1M in THF) was added, and the reaction mixture was allowed to stir for 30 minutes. Geranyl bromide (300 mg, 1.38 mmol) was then added and the reaction was allowed to stir at room temperature overnight. Reaction progress was monitored by analysis of the ^{31}P NMR spectrum. Once it was complete, water was added to quench the reaction. The resulting solution was then extracted with EtOAc and washed with brine. The organic layer was dried (Na_2SO_4) and concentrated in vacuo, and the residue was purified by column chromatography (5% EtOH in hexane) to afford compound **10** as a colorless oil (220 mg, 51%): ^{1}H NMR (300 MHz, $CDCl_3$) δ 5.50 (t, J = 6.7 Hz, 1H), 5.34 (t, J = 5.6 Hz, 1H), 5.16–5.05 (m, 2H), 4.37 (d, J = 6.8 Hz, 2H), 4.30–4.17 (m, 8H), 2.98–

2.82 (m, 2H), 2.16–1.98 (m, 8H), 1.68 (s, 12H), 1.61 (s, 6H), 1.35 (t, J = 6.9 Hz, 6H), 1.35 (t, J = 6.9 Hz, 6H); ^{13}C NMR (75 MHz, $CDCl_3$) δ 139.6, 136.7, 131.4, 131.2, 124.3, 123.9, 120.8, 117.8 (t, J_{CP} = 7.9 Hz), 80.7 (t, J_{CP} = 151.0 Hz), 63.2 (t, J_{CP} = 3.6 Hz 3C), 62.9 (t, J_{CP} = 3.7 Hz, 2C), 40.0, 39.5, 30.0, 26.6, 26.3, 25.6 (2C), 17.6 (2C), 16.5 (t, J_{CP} = 3.0 Hz, 2C), 16.4 (t, J_{CP} = 2.5 Hz, 2C), 16.4, 16.3; ^{31}P NMR (121 MHz, $CDCl_3$) δ 19.0; HRMS (ES$^+$, m/z) calcd for $(M+Na)^+$ $C_{29}H_{54}O_7NaP_2$: 599.3243; found: 599.3244.

Bisphosphonate salt 11. Prepared according to the general procedure given for compound **20**: yield, 17%; white solid; ^{1}H NMR (300 MHz, D_2O) δ 5.49 (t, J = 6.6 Hz, 1H), 5.29–5.21 (m, 1H), 4.32 (d, J = 7.1 Hz, 2H), 3.67 (t, J_{HP} =15.2 Hz, 1H), 2.25–2.08 (m, 4H), 1.74 (s, 3H), 1.72 (s, 3H), 1.66 (s, 3H); ^{13}C NMR (75 MHz, D_2O) δ 142.1, 133.8, 124.3, 120.8, 75.7 (t, J_{CP} = 130.3 Hz), 69.8, 39.0, 25.8, 24.9, 17.0, 15.8; ^{31}P NMR (121 MHz, D_2O) δ 14.1; HRMS (ES$^-$, m/z) calcd for $(M-H)^-$ $C_{11}H_{21}O_7P_2$: 327.0763; found: 327.0748.

Bisphosphonate salt 6. Prepared according to the general procedure given for compound **20**: yield, 20%; white solid; ^{1}H NMR (500 MHz, D_2O) δ 5.65 (t, J = 6.5 Hz, 1H), 5.39 (t, J = 6.2 Hz, 1H), 5.27–5.18 (m, 2H), 4.32 (d, J = 6.9 Hz, 2H), 2.88 (td, J_{HP} = 14.1 Hz, J = 6.5 Hz, 2H), 2.19–2.12 (m, 4H), 2.11–2.05 (m, 4H), 1.70 (s, 6H), 1.69 (s, 6H), 1.65 (s, 3H), 1.64 (s, 3H); ^{13}C NMR (125 MHz, D_2O) δ 141.2, 137.1, 133.7, 133.5, 125.2, 124.7, 121.3, 119.7 (t, J_{CP} = 7.8 Hz), 79.5 (t, J_{CP} = 131.8 Hz), 62.7 (t, J_{CP} = 6.5 Hz), 39.4, 38.9, 28.7, 26.1, 25.7, 25.0, 18.5, 17.1, 17.1, 15.9, 15.6; ^{31}P NMR (201 MHz, D_2O) 17.5; HRMS (ES$^-$, m/z) calcd for $(M-H)^-$ $C_{21}H_{37}O_7P_2$: 463.2015; found: 463.2021.

Monophosphonate ether 12. Yield, 77%; colorless oil; ^{1}H NMR (300 MHz, $CDCl_3$) δ 5.36–5.28 (m, 1H), 4.23–4.11 (m, 4H), 4.10 (d, J = 7.1 Hz, 2H), 3.74 (d, J_{HP} = 8.4 Hz,

2H), 1.76 (s, 3H), 1.70 (s, 3H), 1.35 (t, J = 7.0 Hz, 6H); ^{13}C NMR (75 MHz, $CDCl_3$) δ 137.8, 119.6, 68.6 (d, J_{CP} = 12.0 Hz), 62.7 (d, J_{CP} = 166.8 Hz), 61.7 (d, J_{CP} = 6.1 Hz, 2C), 25.2, 17.4, 15.9 (d, J_{CP} = 5.1 Hz, 2C); ^{31}P NMR (121 MHz, $CDCl_3$) δ 21.7.

Bisphosphonate ether 13 [1]. A solution of *n*-butyllithium in hexanes (8.8 mL, 21.2 mmol) was added to a solution of diisopropylamine (2.75 mL, 19.5 mmol) in THF (16 mL) at –78 °C and the reaction was allowed to stir for 30 minutes. Ether **12** (2 g, 8.5 mmol) was then added to the reaction mixture dropwise (over 90 minutes), allowed to react for one additional hour, and then followed by the careful addition of diethyl chlorophosphate (2.9 mL, 19.5 mmol). After it was allowed to warm to room temperature slowly and to stir overnight, the reaction was quenched by addition of water. The aqueous layer was extracted with EtOAc, and the combined organic layers were dried (Na_2SO_4) and concentrated in vacuo. The resulting residue was purified by column chromatography (5% EtOH in hexane) to afford the desired product **13** as a colorless oil (1.39 g, 44%): ^{1}H NMR (300 MHz, $CDCl_3$) δ 5.34 (t, J = 7.2 Hz, 1H), 4.36–4.08 (m, 8H), 4.32 (d, J = 7.1 Hz, 2H), 4.03 (t, J_{HP} = 17.5 Hz, 1H), 1.77 (s, 3H), 1.72 (s, 3H), 1.37 (t, J = 7.3 Hz, 12H); ^{13}C NMR (75 MHz, $CDCl_3$) δ 139.1, 119.3, 70.1 (t, J_{CP} = 156.9 Hz), 69.4 (t, J_{CP} = 5.2 Hz), 62.9 (t, J_{CP} = 2.6 Hz, 2C), 62.7 (t, J_{CP} = 3.2 Hz, 2C), 25.4, 17.6, 16.1 (t, J_{CP} = 2.9 Hz, 2C), 16.0 (t, J_{CP} = 3.6 Hz, 2C); ^{31}P NMR (121 MHz, $CDCl_3$) δ 16.2; HRMS (ES^+, *m*/*z*) calcd for $(M+Na)^+$ $C_{14}H_{30}O_7NaP_2$: 395.1365; found: 395.1395.

Bisphosphonate salt 14. Yield, 73%; white solid; ^{1}H NMR (300 MHz, D_2O) δ 5.38 (t, J = 6.7 Hz, 1H), 4.25 (d, J = 7.2 Hz, 2H), 3.67 (t, J_{HP} = 16.2 Hz, 1H), 1.74 (s, 3H), 1.68 (s, 3H); ^{13}C NMR (100 MHz, D_2O) δ 140.2, 119.9, 74.1 (t, J_{CP} = 140.6 Hz), 69.8, 25.1, 17.5;

^{31}P NMR (121 MHz, D_2O) δ 13.9; HRMS (ES$^-$, *m/z*) calcd for $(M-H)^-$ $C_6H_{13}O_7P_2$: 259.0137; found: 259.0145.

Bisphosphonate ester 15. Yield, 37%; colorless oil; ^1H NMR (300 MHz, $CDCl_3$) δ 5.48 (t, J = 6.2 Hz, 1H), 5.31 (t, J = 6.7 Hz, 1H), 5.16–5.04 (m, 1H), 4.33 (d, J = 6.8 Hz, 2H), 4.30–4.10 (m, 8H), 2.89 (td, J_{HP} = 14.5 Hz, J = 6.4 Hz, 2H), 2.15–1.96 (m, 4H), 1.72 (s, 3H), 1.68 (s, 3H), 1.67 (s, 3H), 1.65 (s, 3H), 1.60 (s, 3H), 1.34 (t, J = 7.0 Hz, 6H), 1.34 (t, J = 6.8 Hz, 6H); ^{13}C NMR (100 MHz, $CDCl_3$) δ 136.8, 136.5, 131.4, 124.4, 121.2, 117.9 (t, J_{CP} = 8.1 Hz), 80.8 (t, J_{CP} = 150.6 Hz), 63.4 (t, J_{CP} =3.2 Hz), 63.4 (t, J_{CP} =4.5 Hz, 2C), 63.0 (t, J_{CP} = 3.6 Hz, 2C), 40.1, 30.2, 26.7, 25.8, 25.8, 18.2, 17.7, 16.6 (t, J_{CP} = 2.4 Hz, 2C), 16.6 (t, J_{CP} = 3.0 Hz, 2C), 16.5; ^{31}P NMR (121 MHz, $CDCl_3$) δ 19.1; HRMS (ES$^+$, *m/z*) calcd for $(M+H)^+$ $C_{24}H_{47}O_7P_2$: 509.2797; found: 509.2803.

Bisphosphonate salt 16. Yield, 33%; white solid; ^1H NMR (500 MHz, D_2O) δ 5.65 (t, J = 6.3 Hz, 1H), 5.39 (t, J = 6.6 Hz, 1H), 5.25 (t, J = 6.0 Hz, 1H), 4.31 (d, J = 7.0 Hz, 2H), 2.87 (td, J_{HP} = 13.3 Hz, J = 6.4 Hz, 2H), 2.20–2.12 (m, 2H), 2.11-2.05 (m, 2H), 1.75 (s, 3H), 1.70 (s, 6H), 1.69 (s, 3H), 1.65 (s, 3H); ^{13}C NMR (125 MHz, D_2O) δ 138.7, 137.1, 133.6, 124.7, 121.1, 119.7 (t, J_{CP} = 7.8 Hz), 79.5 (t, J_{CP} = 131.7 Hz), 62.7 (t, J_{CP} = 6.0 Hz), 39.3, 28.9, 26.0, 25.0, 24.9, 17.5, 17.1, 15.5; ^{31}P NMR (201 MHz, D_2O) δ 17.5; HRMS (ES$^-$, *m/z*) calcd for $(M-H)^-$ $C_{16}H_{29}O_7P_2$: 395.1389; found: 395.1400.

Bisphosphonate ester 17. Yield, 29%; colorless oil; ^1H NMR (500 MHz, $CDCl_3$) δ 5.45 (t, J = 6.5 Hz, 1H), 5.31 (tt, J = 6.9 HZ, J_{HP} = 1.4 Hz, 1H), 4.33 (d, J = 6.7 Hz, 2H), 4.28–4.18 (m, 8H), 2.87 (td, J_{HP} = 14.7 Hz, J = 6.8 Hz, 2H), 1.73 (s, 6H), 1.67 (s, 3H), 1.65 (s, 3H), 1.34 (t, J = 7.6 Hz, 6H), 1.34 (t, J = 7.2 Hz, 6H); ^{13}C NMR (125 MHz, $CDCl_3$) δ 136.6, 133.3, 121.2, 118.1 (t, J_{CP} = 7.8 Hz), 80.8 (t, J_{CP} = 150.5 Hz), 63.5 (t, J_{CP} = 5.2

Hz), 63.3 (t, J_{CP} = 3.2 Hz, 2C), 63.1 (t, J_{CP} = 3.7 Hz, 2C), 30.4, 26.1, 25.8, 18.2, 18.1, 16.6 (t, J_{CP} = 2.6 Hz, 4C); ^{31}P NMR (201 MHz, $CDCl_3$) δ 19.0; HRMS (ES^+, *m/z*) calcd for $(M+Na)^+$ $C_{19}H_{38}O_7NaP_2$: 463.1991; found: 463.1989.

Bisphosphonate salt 18. Yield, 87%; white solid; ^{1}H NMR (500 MHz, D_20) δ 5.70 (s, 1H), 5.39 (t, *J* = 5.9 HZ, 1H), 4.30 (d, *J* = 6.7 Hz, 2H), 2.82 (td, J_{HP} = 12.4 Hz, *J* = 5.7 Hz, 2H), 1.75 (s, 3H) 1.74 (s, 3H), 1.69 (s, 3H), 1.68 (s, 3H); ^{13}C NMR (125 MHz, D_2O) δ 137.9, 132.7, 121.7, 121.5 (t, J_{CP} = 7.7 Hz), 80.4 (t, J_{CP} = 127.1 Hz), 62.4 (t, J_{CP} =5.5 Hz), 30.3, 25.3, 25.1, 17.5, 17.4; ^{31}P NMR (201 MHz, D_2O) δ 17.7; HRMS (ES^-, *m/z*) calcd for $(M-H)^-$ $C_{11}H_{21}O_7P_2$: 327.0763; found: 327.0780.

Bisphosphonate ester 19. Yield, 30%; colorless oil; ^{1}H NMR (300 MHz, $CDCl_3$) δ 5.45 (t, *J* = 6.9 Hz, 1H), 5.33 (t, *J* = 6.5 Hz, 1H), 5.13–5.04 (m, 1H), 4.37 (d, *J* = 6.5 Hz, 2H), 4.30–4.16 (m, 8H), 2.88 (td, J_{HP} = 14.2 Hz, *J* = 6.5 Hz, 2H), 2.15–1.97 (m, 4H), 1.73 (s, 3H), 1.68 (s, 3H), 1.66 (s, 6H), 1.61 (s, 3H), 1.35 (t, *J* = 6.9 Hz, 6H), 1.34 (t, *J* = 7.3 Hz, 6H); ^{13}C NMR (75 MHz, $CDCl_3$) δ 139.7, 133.2, 131.6, 124.0, 120.8, 118.0 (t, J_{CP} = 7.5 Hz), 80.7 (t, J_{CP} = 150.9 Hz), 63.3 (t, J_{CP} = 5.9 Hz), 63.3 (t, J_{CP} = 3.2 Hz, 2C), 63.0 (t, J_{CP} = 3.0 Hz, 2C), 39.5, 30.2, 26.4, 26.0, 25.7, 18.1, 17.7, 16.5 (t, J_{CP} = 3.2 Hz, 4C), 16.5; ^{31}P NMR (121 MHz, $CDCl_3$) δ 19.0; HRMS (ES^+, *m/z*) calcd for $(M+Na)^+$ $C_{24}H_{46}O_7P_2Na$: 531.2617; found: 531.2619.

Bisphosphonate salt 20. 2,4,6-Collidine (0.22 mL, 1.67 mmol) was added to an ice cold solution of bisphosphonate **19** (85 mg, 0.17 mmol) in CH_2Cl_2 (5 mL) followed by the addition of excess TMSBr (0.27 mL, 2.00 mmol). The reaction was allowed to warm slowly to rt and allowed to stir overnight. Once the reaction was complete based on analysis of the ^{31}P NMR spectrum, the volatile materials were removed in vacuo. The

resulting residue was washed with toluene and concentrated repeatedly to remove any remaining TMSBr. It was treated with NaOH (0.27 mL 5M NaOH, 2 mL H_2O) for 10 minutes and then the water was removed on a lyophilizer to obtain the crude salt. This material was precipitated from water by addition of acetone to obtain the desired product, the salt **20** as a white solid (77 mg, 94%): ^{1}H NMR (500 MHz, D_2O) δ 5.84 (s, 1H), 5.39 (t, J = 6.8 Hz, 1H), 5.22–5.17 (m, 1H), 4.17 (d, J = 7.0 Hz, 2H), 2.89–2.79 (m, 2H), 2.16–2.03 (m, 4H), 1.72 (s, 3H), 1.69 (s, 3H), 1.68 (s, 3H), 1.65 (s, 3H), 1.62 (s, 3H); ^{13}C NMR (125 MHz, D_2O) δ 140.6, 133.7, 131.4, 124.2, 123.1 (t, J_{CP} = 6.4 Hz), 122.4, 82.4 (t, J_{CP} = 134.7 Hz), 61.1 (t, J_{CP} = 6.2 Hz), 39.1, 29.7, 25.9, 25.4, 25.0, 17.4, 17.1, 15.6; ^{31}P NMR (201 MHz, D_2O) δ 17.8; HRMS (ES^-, *m*/*z*) calcd for $(M-H)^-$ $C_{16}H_{29}O_7P_2$: 395.1389; found: 395.1388.

Monophosphonate ether 21. Yield, 34%; colorless oil; ^{1}H NMR (500 MHz, $CDCl_3$) δ 5.10–5.05 (m, 1H), 4.20–4.12 (m, 4H), 3.75 (dd, J_{HP} = 8.7 Hz, J = 1.9 Hz, 2H), 3.63–3.57 (m, 2H), 2.05–1.89 (m, 2H), 1.70–1.53 (m, 2H), 1.66 (s, 3H), 1.59 (s, 3H), 1.43–1.28 (m, 2H), 1.34 (t, J = 7.4 Hz, 6H), 1.23–1.12 (m, 1H), 0.90 (d, J = 6.7 Hz, 3H); ^{13}C NMR (125 MHz, $CDCl_3$) δ 130.1, 124.0, 71.2 (d, J_{CP} = 11.5 Hz), 64.3 (d, J_{CP} = 165.6 Hz), 61.4 (d, J_{CP} = 5.7 Hz, 2C), 36.4, 35.7, 28.6, 24.9, 24.7, 18.7, 16.8, 15.7 (d, J_{CP} = 5.4 Hz, 2C); ^{31}P NMR (201 MHz, $CDCl_3$) δ 21.0; HRMS (ES^+, *m*/*z*) calcd for $(M+H)^+$ $C_{15}H_{32}O_4P$: 307.2038; found: 307.2044.

Bisphosphonate ether 22. Yield, 53%; colorless oil; ^{1}H NMR (400 MHz, $CDCl_3$) δ 5.12–5.05 (m, 1H), 4.30–4.20 (m, 8H), 3.91 (t, J_{HP} = 17.6 Hz, 1H), 3.85–3.75 (m, 2H), 2.06–1.88 (m, 2H), 1.73–1.49 (m, 2H), 1.67 (s, 3H), 1.60 (s, 3H), 1.47–1.25 (m, 2H), 1.36 (t, J = 7.0 Hz, 12H), 1.23–1.12 (m, 1H), 0.91 (d, J = 6.7 Hz, 3H); ^{13}C NMR (100

MHz, $CDCl_3$) δ 130.5, 124.3, 73.2 (t, J_{CP} = 157.0 Hz), 73.0 (t, J_{CP} =4.6 Hz), 62.9 (t, J_{CP} = 3.1 Hz), 62.8 (t, J_{CP} = 3.3 Hz), 62.7 (t, J_{CP} = 3.5 Hz), 62.7 (t, J_{CP} = 3.2 Hz), 36.7, 36.4, 28.8, 25.2, 25.0, 19.0, 17.2, 16.1 (t, J_{CP} = 3.6 Hz, 2C), 16.0 (t, J_{CP} = 3.1 Hz, 2C); ^{31}P NMR (121 MHz, $CDCl_3$) δ 15.8; HRMS (ES$^+$, *m*/*z*) calcd for $(M+H)^+$ $C_{19}H_{41}O_7P_2$: 443.2328; found: 443.2325.

Tetraethyl bisphosphonate ether 23. Yield, 45%; colorless oil; ^{1}H NMR (400 MHz, $CDCl_3$) δ 5.46 (t, *J* = 6.3 Hz, 1H), 5.15–5.05 (m, 2H), 4.29–4.16 (m, 8H), 3.87–3.78 (m, 2H), 2.79 (td, J_{HP} = 14.8 Hz, *J* = 6.6 Hz, 2H), 2.14–1.90 (m, 6H), 1.76–1.52 (m, 4H), 1.68 (s, 3H), 1.65 (s, 3H), 1.60 (s, 3H), 1.60 (s, 3H), 1.40–1.30 (m, 15H), 1.22–1.12 (m, 1H), 0.89 (d, *J* = 6.5 Hz, 3H); ^{13}C NMR (100 MHz, $CDCl_3$) δ 136.8, 131.3, 131.1, 124.9, 124.4, 117.8 (t, J_{CP} = 7.9 Hz), 81.0 (t, J_{CP} = 150.4 Hz), 84.7 (t, J_{CP} =5.5 Hz), 63.3 (t, J_{CP} = 2.9 Hz), 63.3 (t, J_{CP} = 3.6 Hz), 62.9 (t, J_{CP} = 3.2 Hz), 62.9 (t, J_{CP} = 4.3 Hz), 40.0, 37.4 (2C), 29.9, 29.4, 26.7, 25.7 (2C), 25.5, 19.6, 17.7, 17.6, 16.6 (t, J_{CP} = 3.3 Hz, 2C), 16.5 (t, J_{CP} = 3.3 Hz, 2C), 16.4; ^{31}P NMR (121 MHz, $CDCl_3$) δ 19.2; HRMS (ES$^+$, *m*/*z*) calcd for $(M+H)^+$ $C_{29}H_{57}O_7P_2$: 579.3580; found: 579.3573.

Bisphosphonate salt 24. Yield, 17%; white solid; ^{1}H NMR (500 MHz, D_20) δ 5.73 (t, *J* = 5.5 Hz, 1H), 5.28–5.22 (m, 2H), 3.85–3.73 (m, 2H), 2.87–2.75 (m, 2H), 2.20–1.95 (m, 6H), 1.71 (s, 3H), 1.70 (s, 3H), 1.67 (s, 3H), 1.65 (s, 3H), 1.64 (s, 3H), 1.61–1.45 (m, 2H), 1.44–1.31 (m, 2H), 1.22–1.12 (m, 1H), 0.89 (d, *J* = 6.4 H, 3H); ^{13}C NMR (125 MHz, D_2O) δ 136.0, 133.5, 133.1, 125.5, 124.9, 121.6, 80.0 (t, J_{CP} = 126.5 Hz), 64.9, 39.5, 37.3, 36.9, 29.4, 26.2, 25.0 (3C), 24.9, 19.2, 17.1, 17.0, 15.5; ^{31}P NMR (201 MHz, D_2O) δ 17.7; HRMS (ES$^-$, *m*/*z*) calcd for $(M-H)^-$ $C_{21}H_{39}O_7P_2$: 465.2171; found: 465.2168.

FDPS and GGDPS enzyme assays. The enzymes FDPS and GGDPS were kindly provided by Dr. James E. Dunford. Both the FDPS and GGPDS assays were implemented with a method modified from Dunford et al. [2]. Enzymes were diluted to 2 µg/mL (10 mM HEPES, pH 7.5, 500 mM NaCl, 5% glycerol, 1 mM TCEP, and 5 µg/mL BSA) and pre incubated with inhibitors in the reaction buffer (50 mM Tris, pH 7.7, 2 mM $MgCl_2$, 0.5 mM TCEP, and 50 µg/mL BSA) for 10 min at room temperature. Both FDPS and GGDPS enzyme assay reactions were initiated by the simultaneous addition of either 10 µM GPP or 10 µM FPP and ^{14}C-IPP and were allowed to proceed at 37 °C for 3 min and 15 min respectively, at which point no more than 20% of the substrate was used. Reactions were terminated by the addition of 200 µL saturated NaCl and isoprenoids were extracted with 1 mL saturated butanol. Incorporated ^{14}C was detected by liquid scintillation counting.

References.

(1) Biller, S. A.; Sofia, M. J.; Abt, J. W.; Delange, B.; Dickson, J. K.; Forster, C.; Gordon, E. M.; Harrity, T.; Magnin, D. R.; Marretta, J.; Rich, L. C.; Ciosek, C. P. *ACS Symp. Ser.* **1992**, *497*, 65-80.

(2) Dunford, J. E.; Thompson, K.; Coxon, F. P.; Luckman, S. P.; Hahn, F. M.; Poulter, C. D.; Ebetino, F. H.; Rogers, M. J. *J. Pharmacol. Exp. Ther.* **2001**, *296*, 235-242.

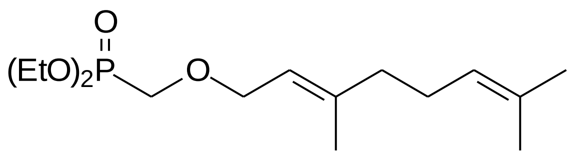

8

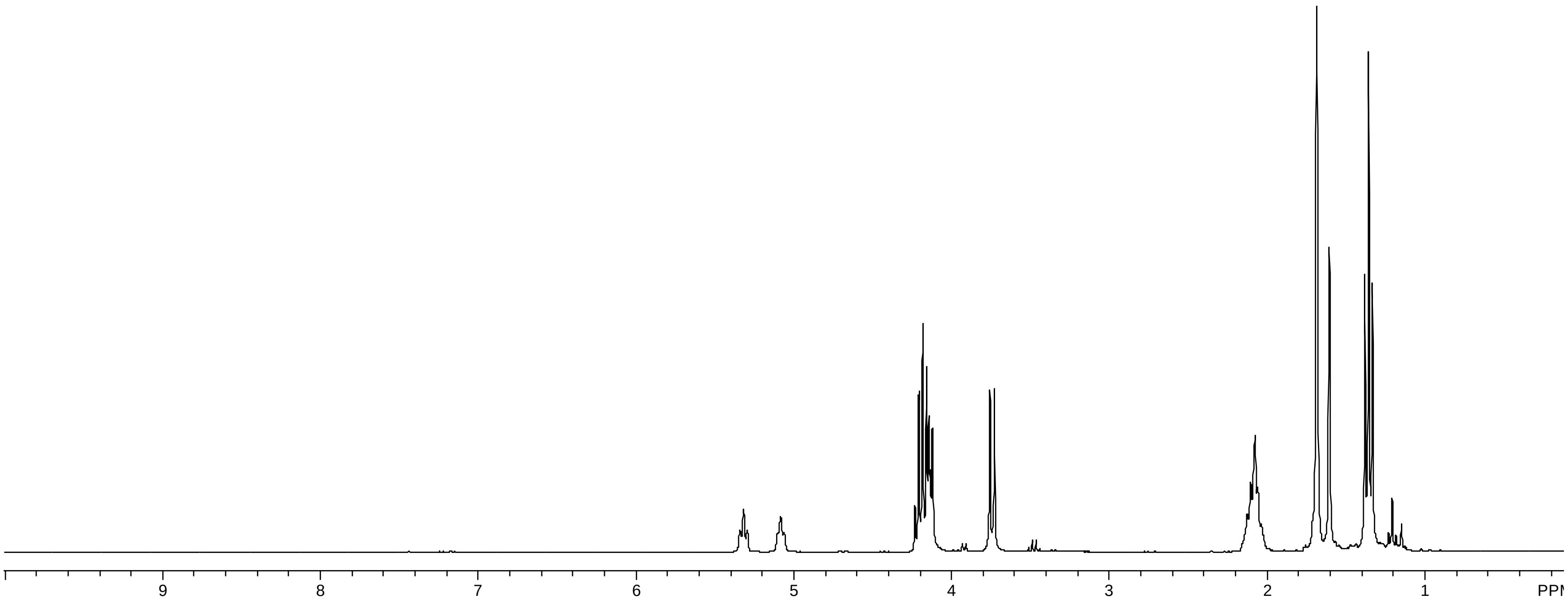

300 MHz ^{1}H NMR Spectrum of Compound **8**.

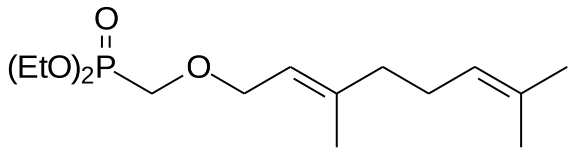

8

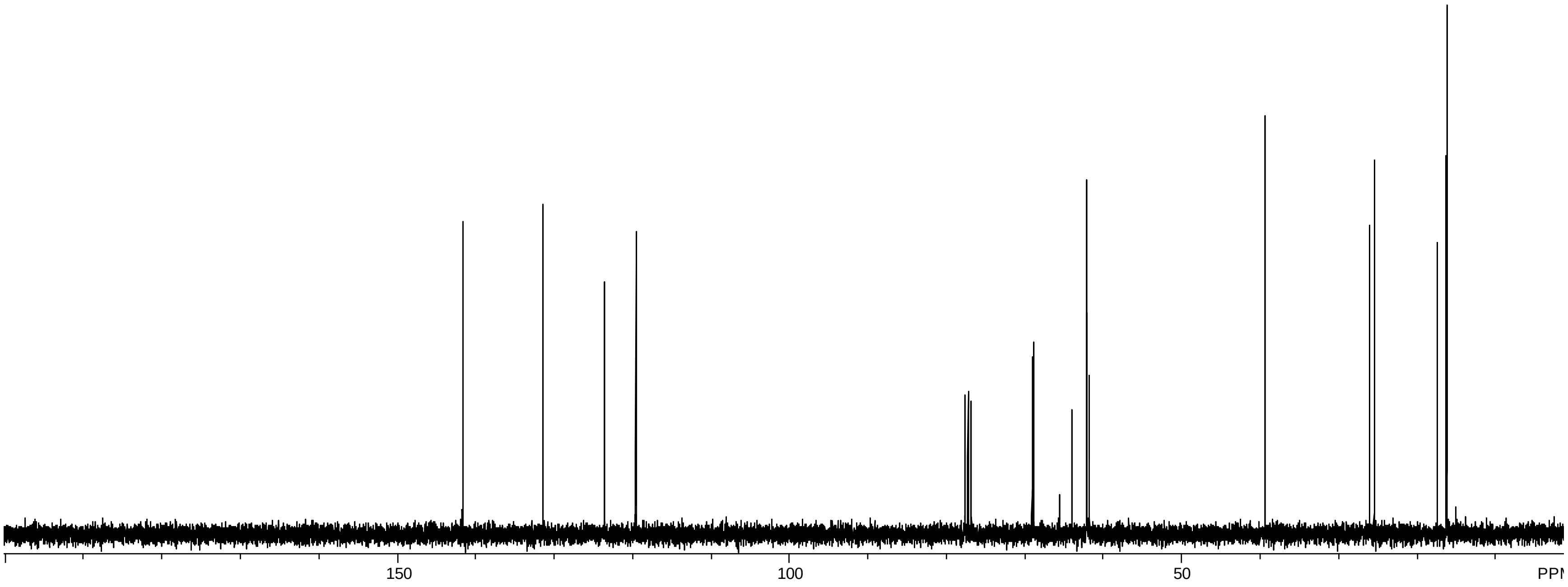

75 MHz ^{13}C NMR Spectrum of Compound **8**.

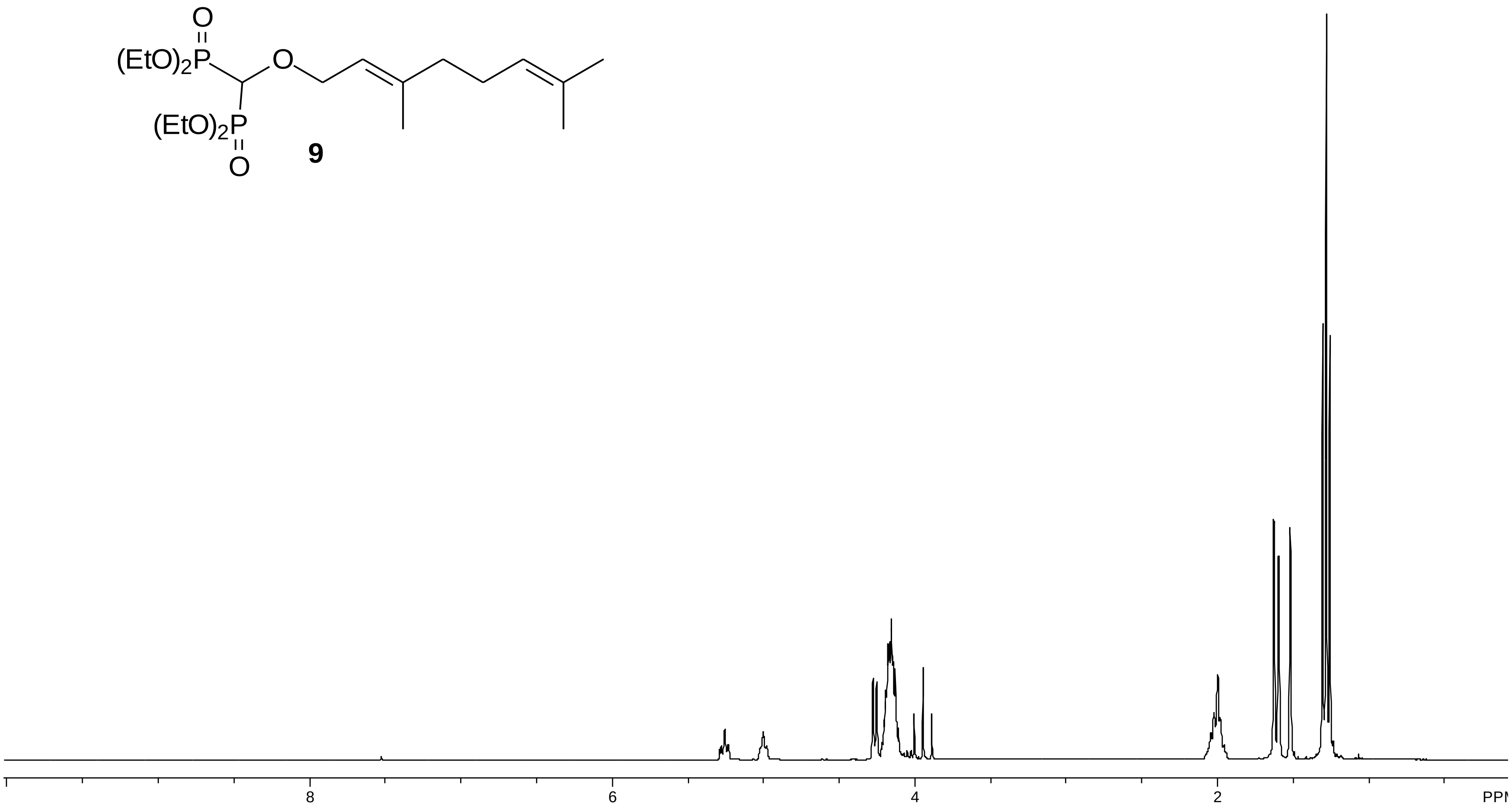

300 MHz ^{1}H NMR Spectrum of Compound **9**.

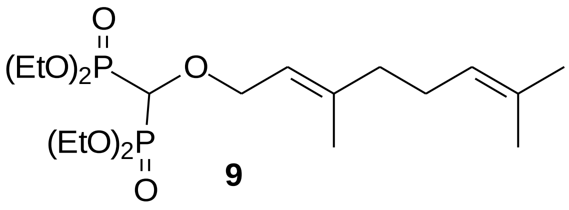

9

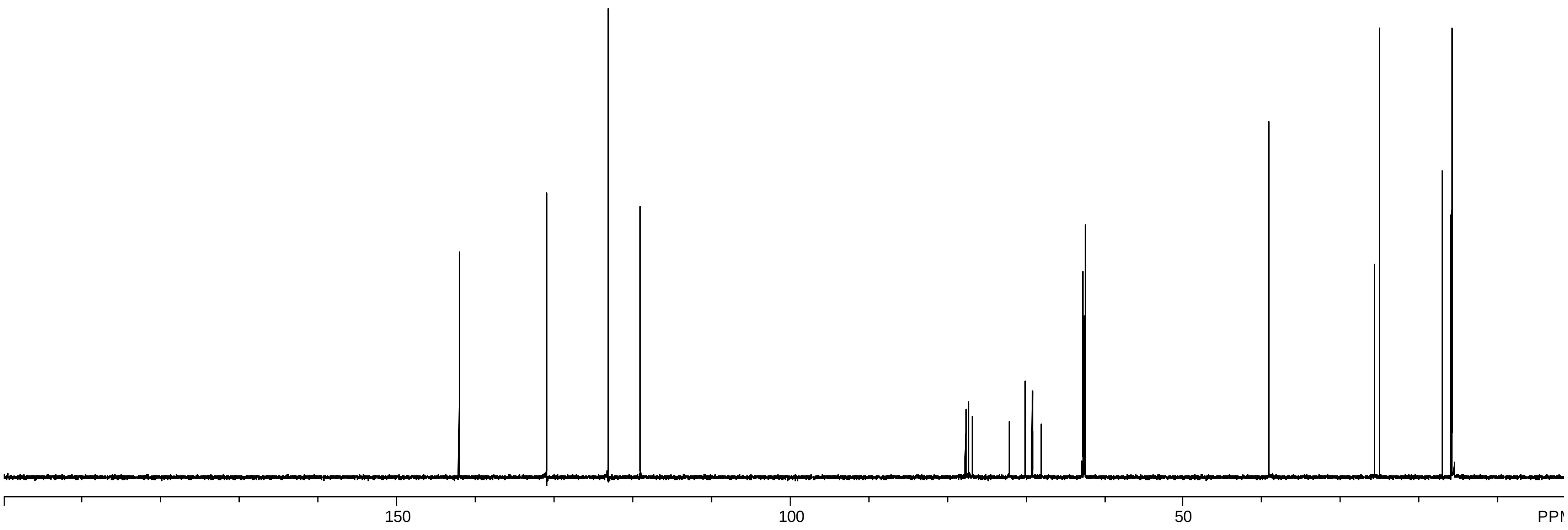

75 MHz ^{13}C NMR Spectrum of Compound **9**.

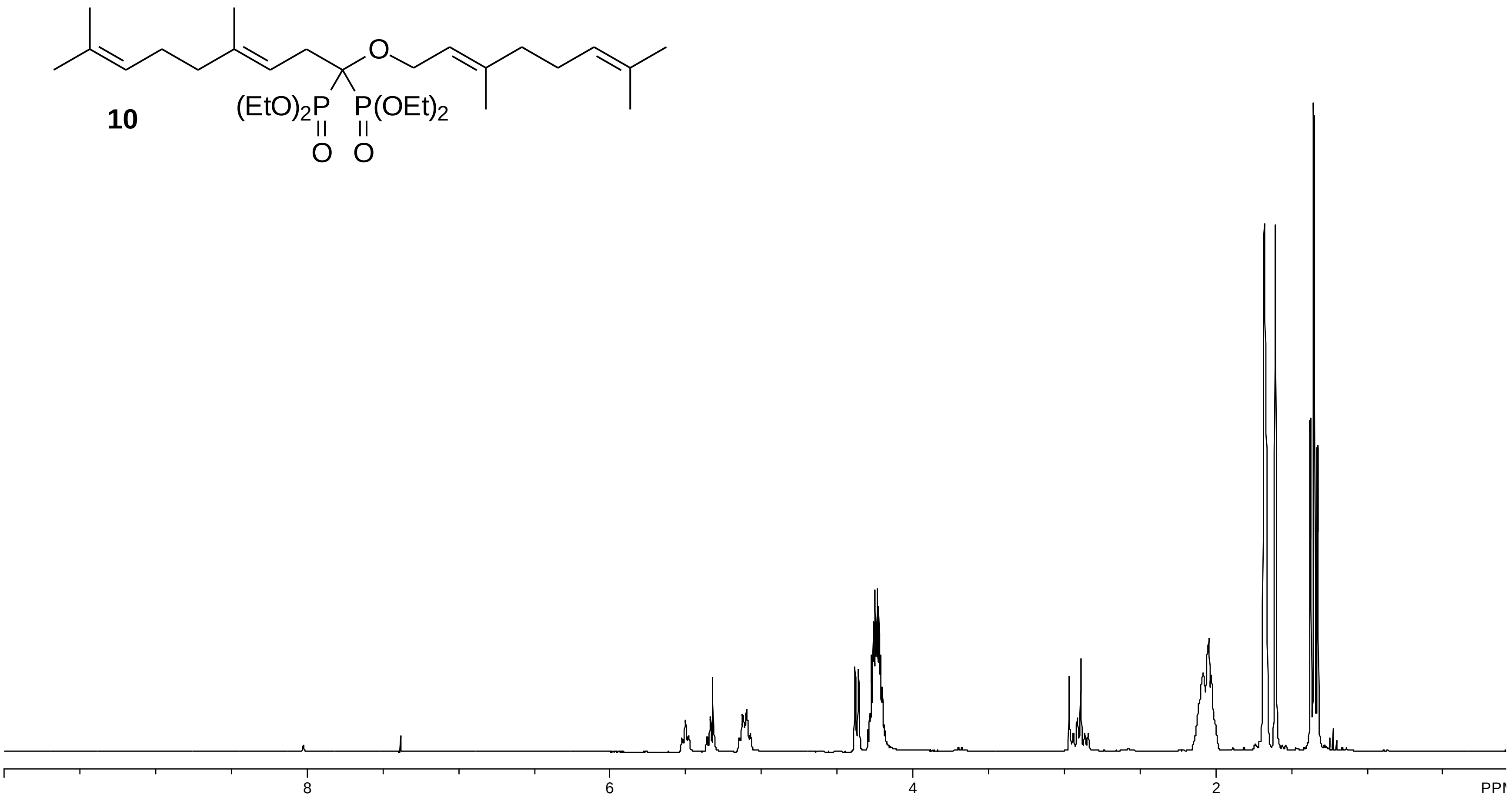

300 MHz ^{1}H NMR Spectrum of Compound **10**.

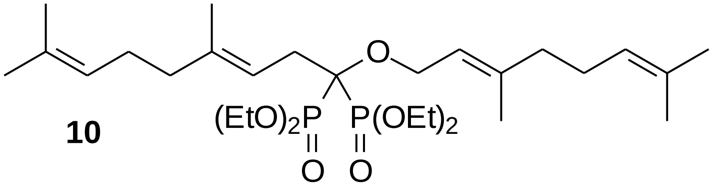

10

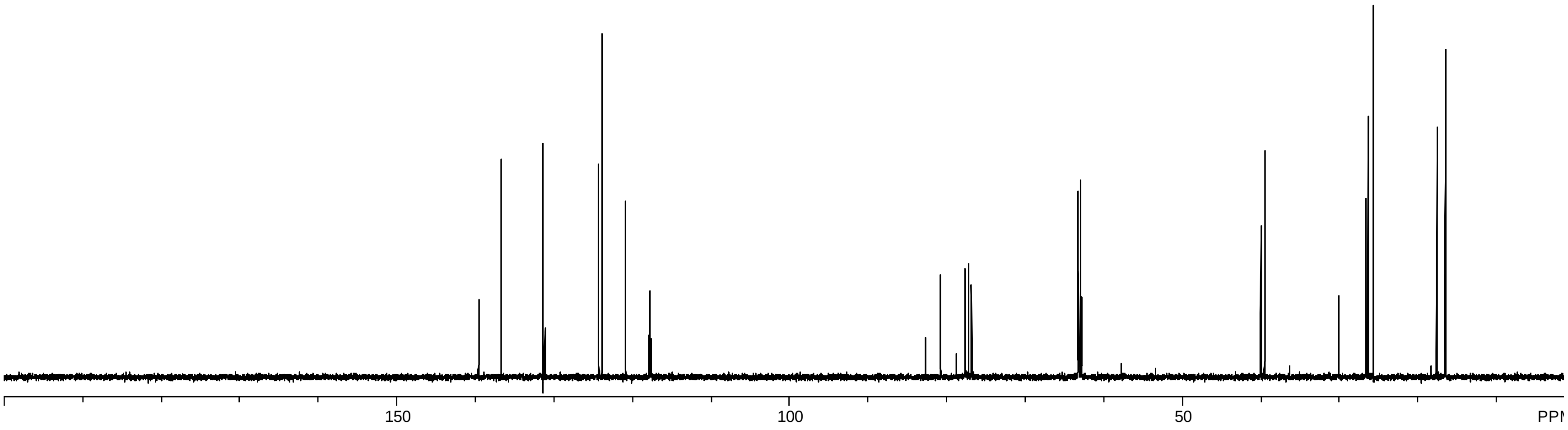

75 MHz ^{1}H NMR Spectrum of Compound **10**.

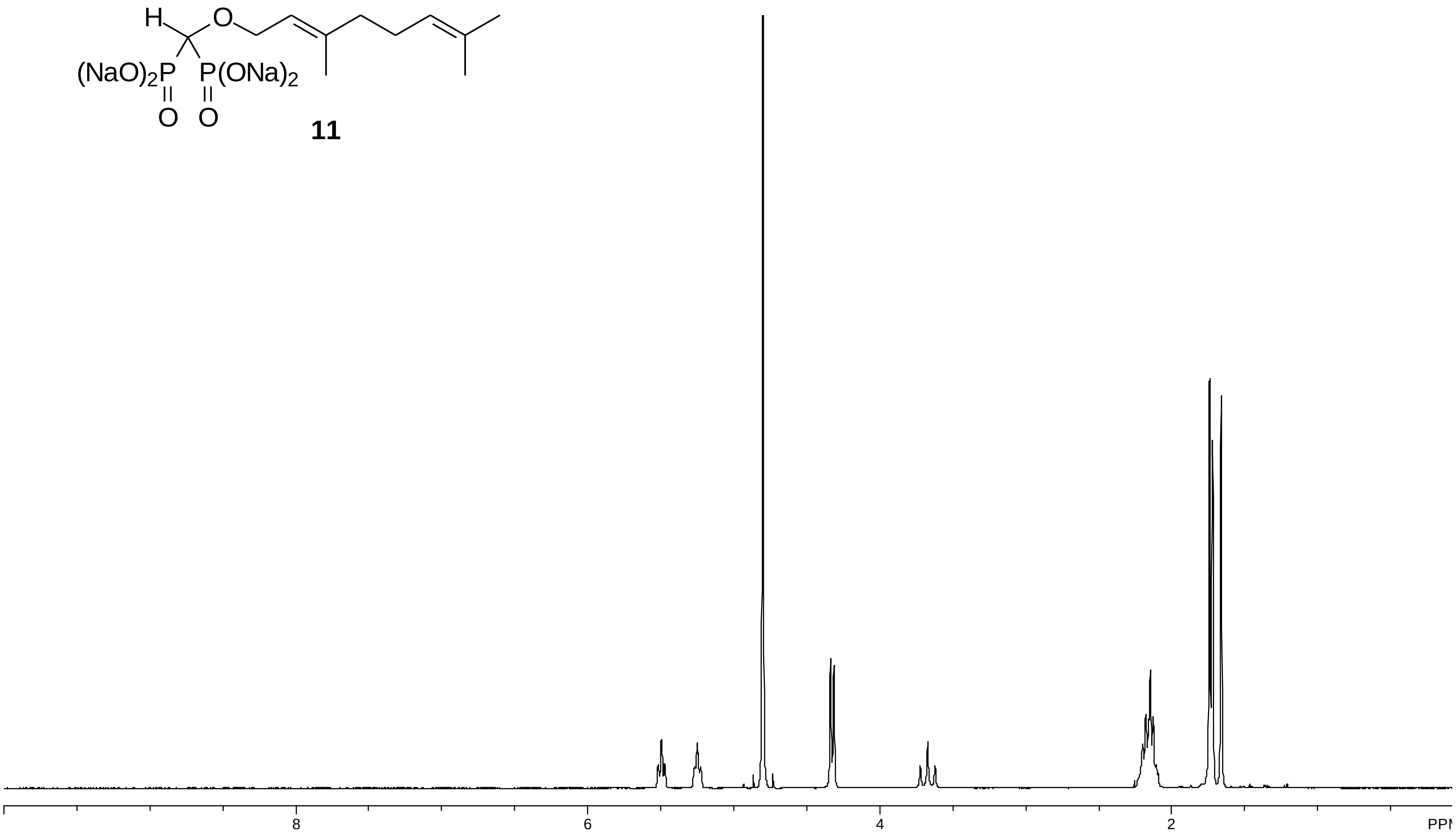

300 MHz ^{1}H NMR Spectrum of Compound **11**.

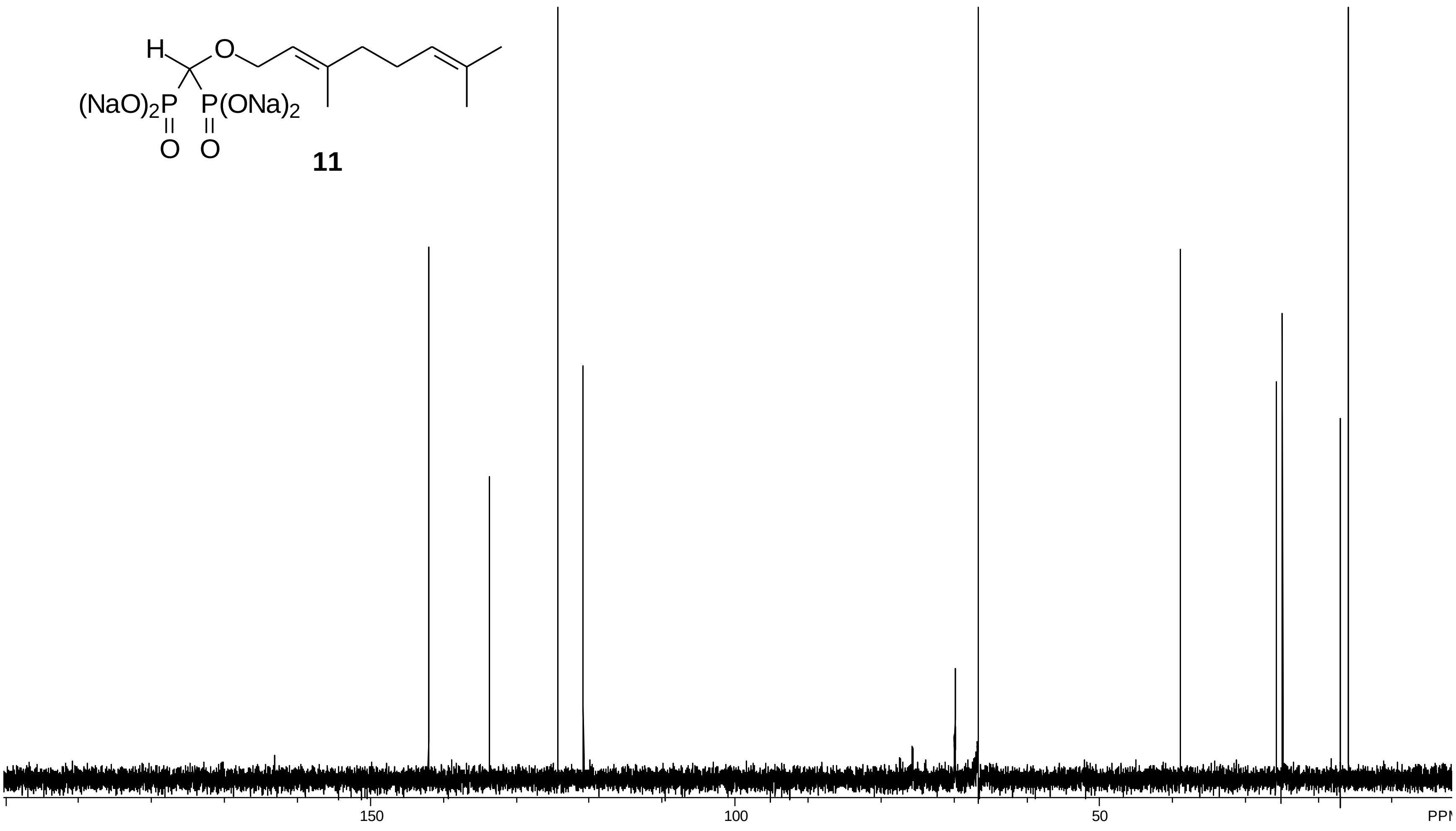

75 MHz ^{1}H NMR Spectrum of Compound **11**.

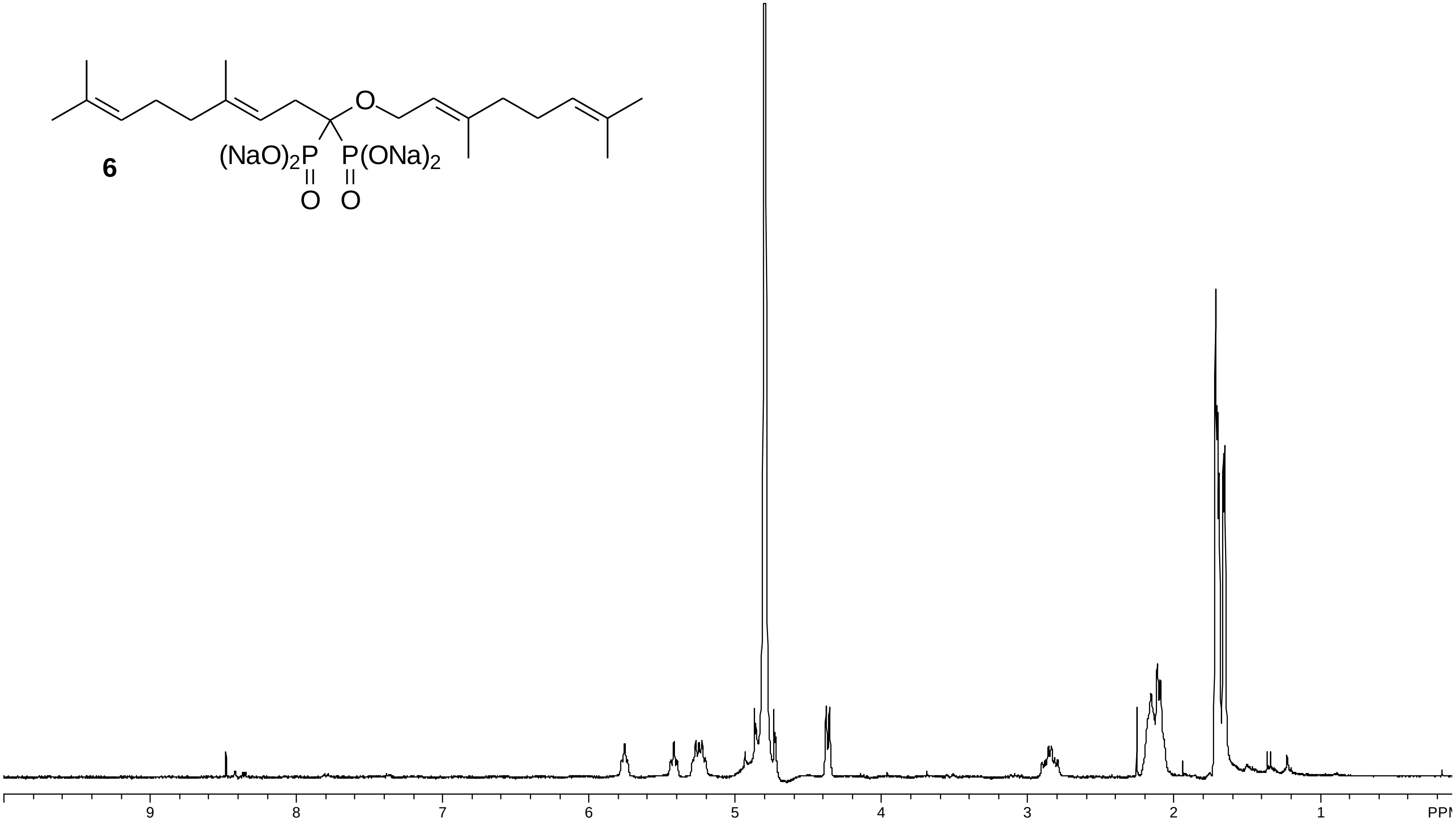

300 MHz ^{1}H NMR Spectrum of Compound **6**.

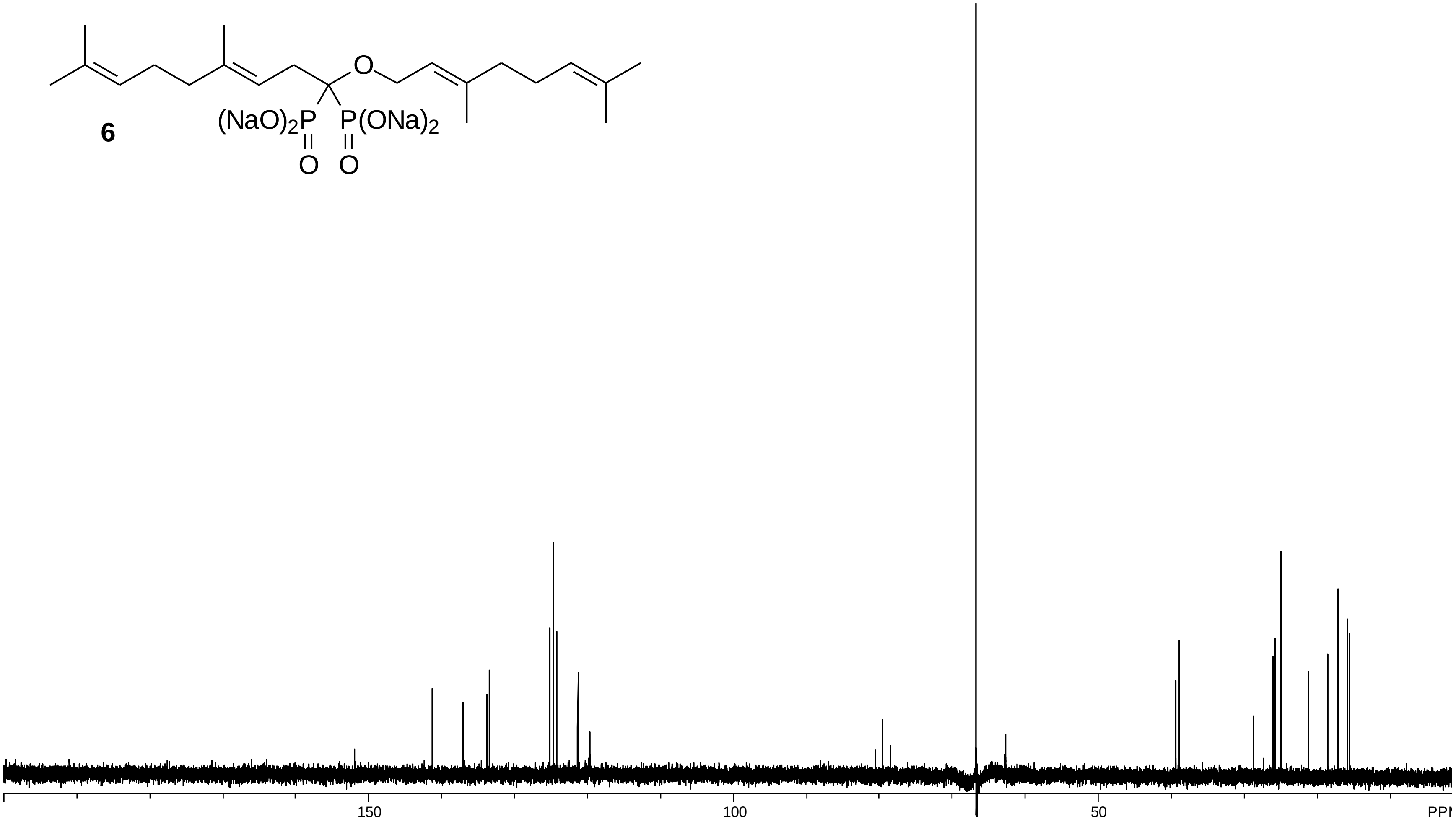

125 MHz ^{13}C NMR Spectrum of Compound **6**.

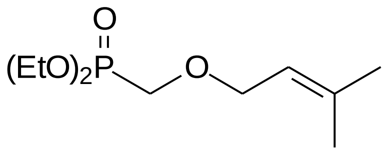

12

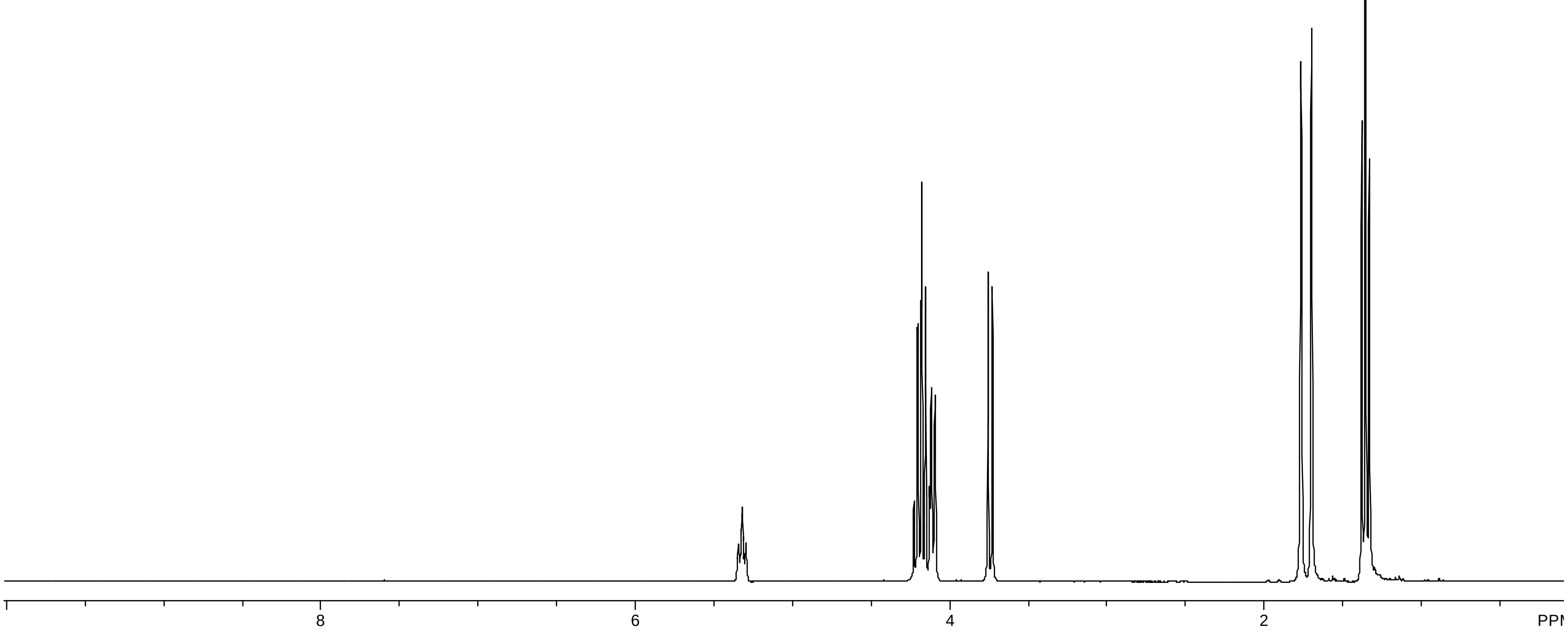

300 MHz ^{1}H NMR Spectrum of Compound **12**.

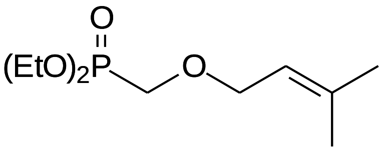

12

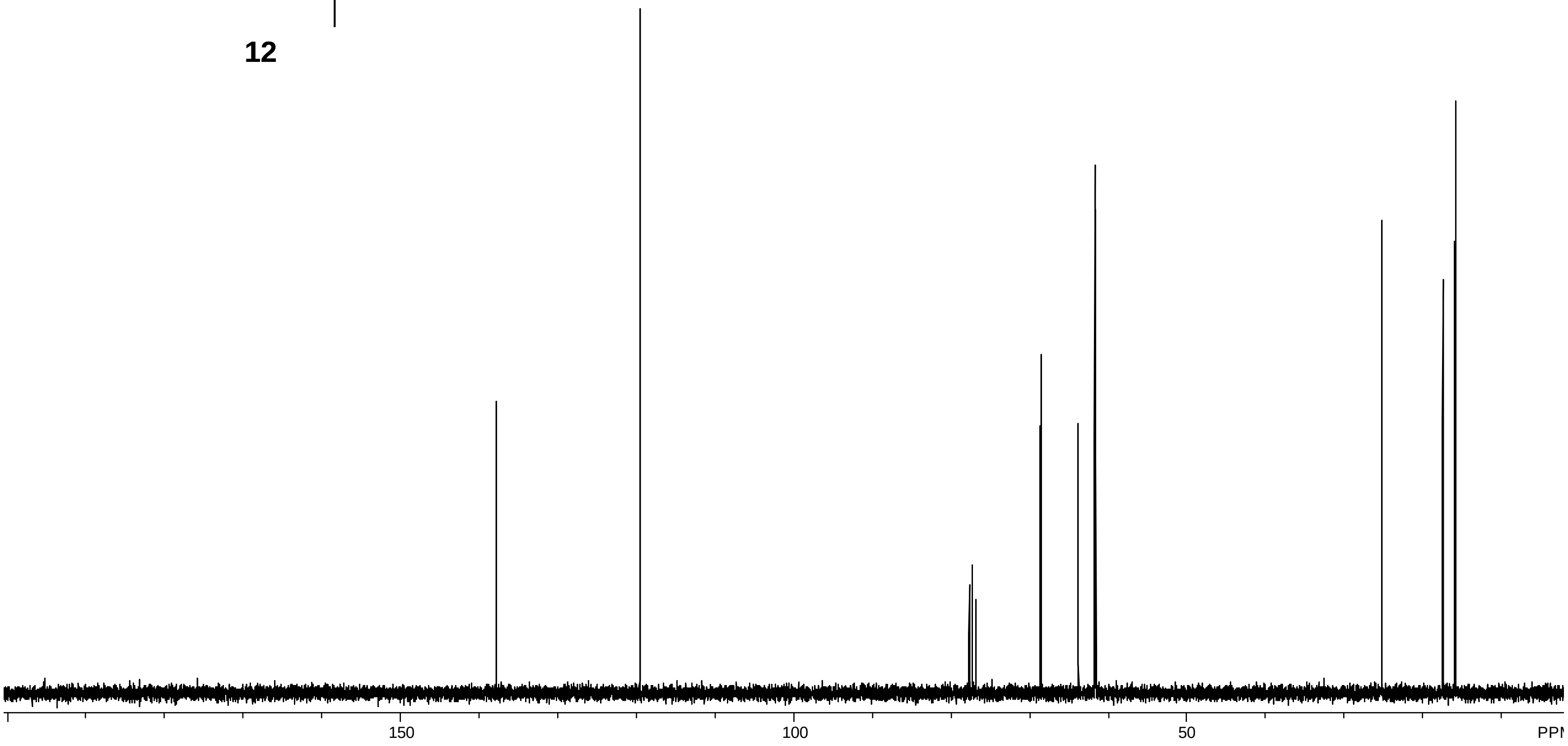

75 MHz ^{13}C NMR Spectrum of Compound **12.**

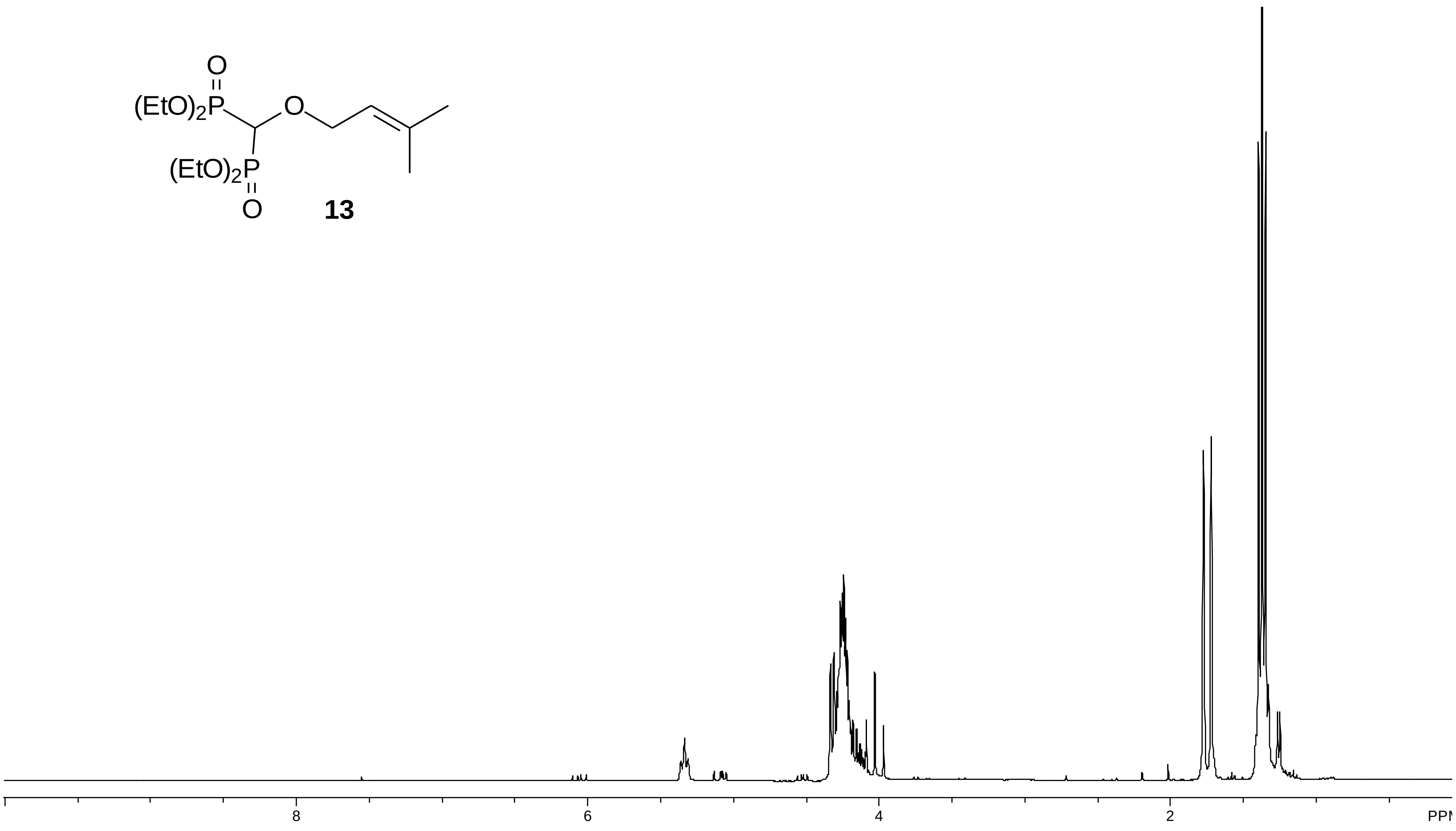

300 MHz ¹H NMR Spectrum of Compound **13**.

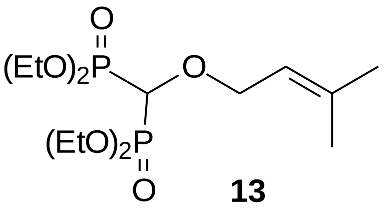

13

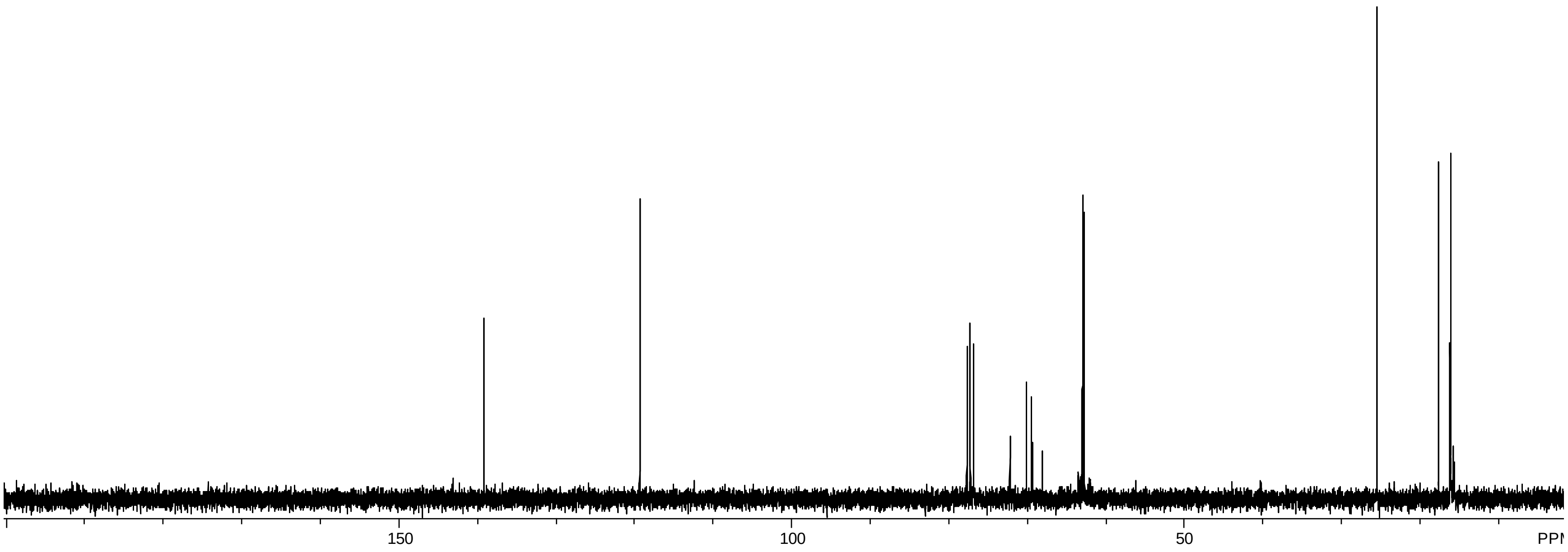

75 MHz ^{13}C NMR Spectrum of Compound **13**.

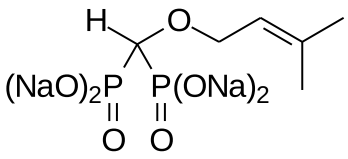

14

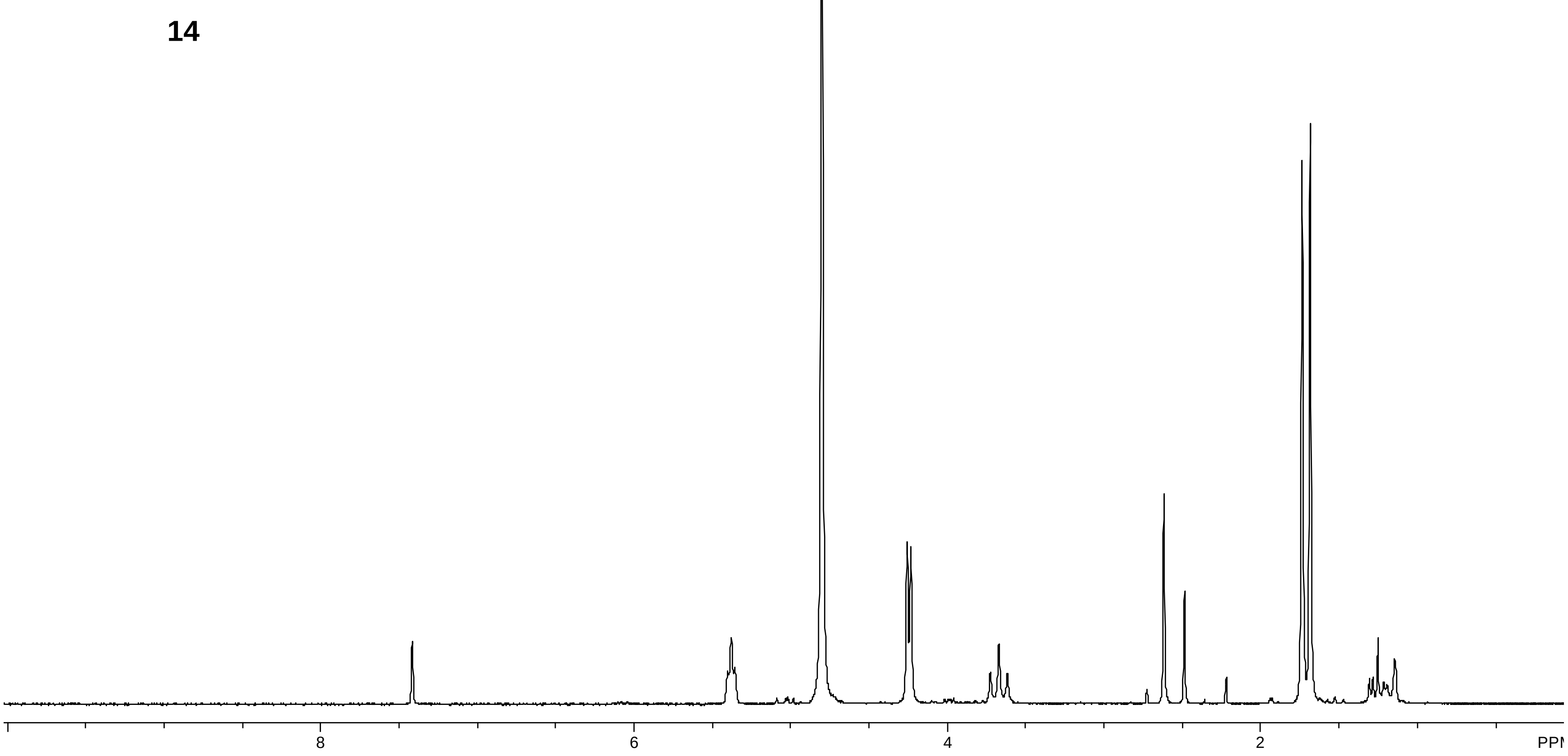

300 MHz ^{1}H NMR Spectrum of Compound **14**.

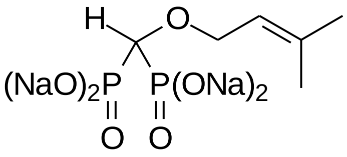

14

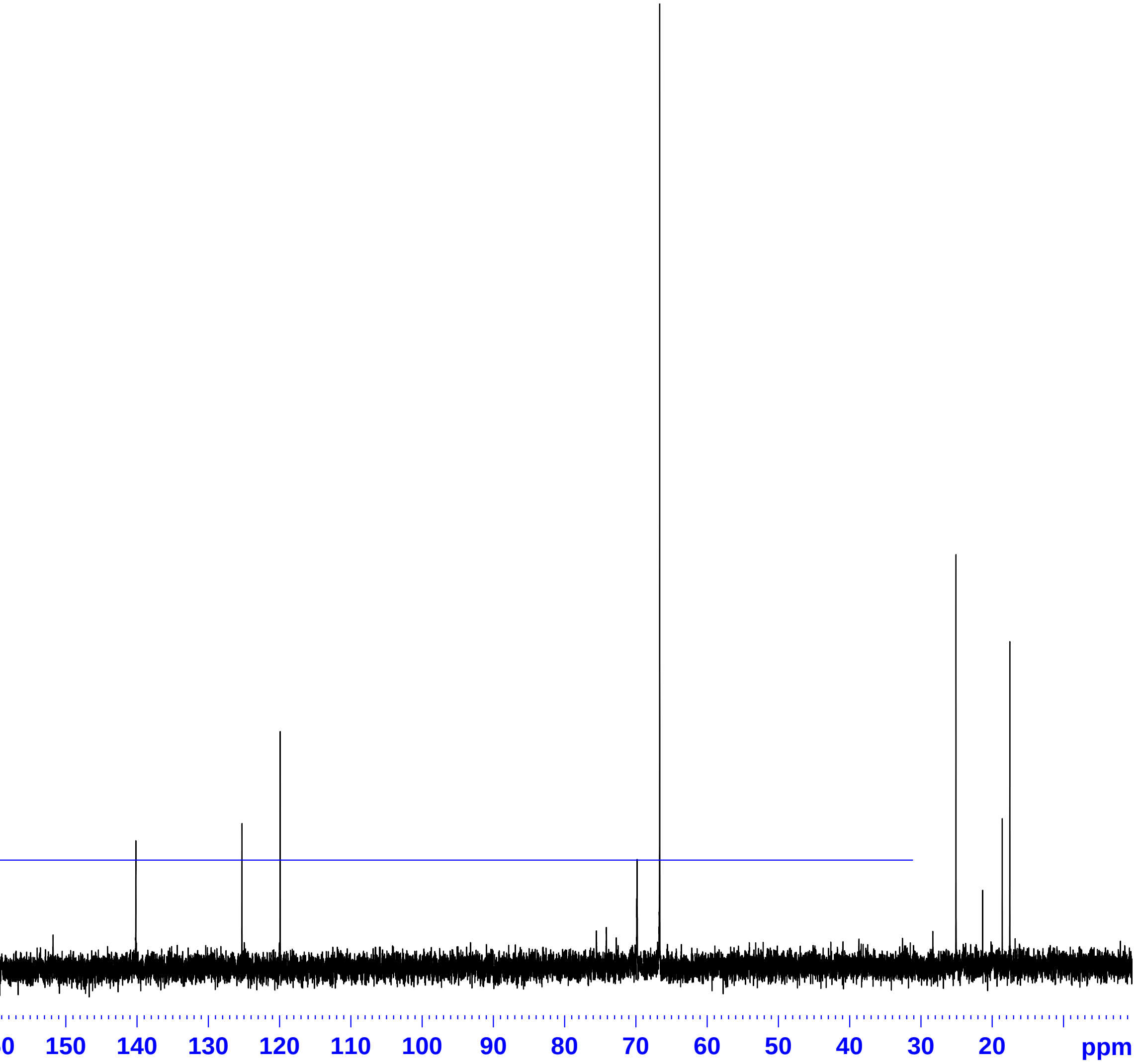

100 MHz ^{13}C NMR Spectrum of Compound **14**.

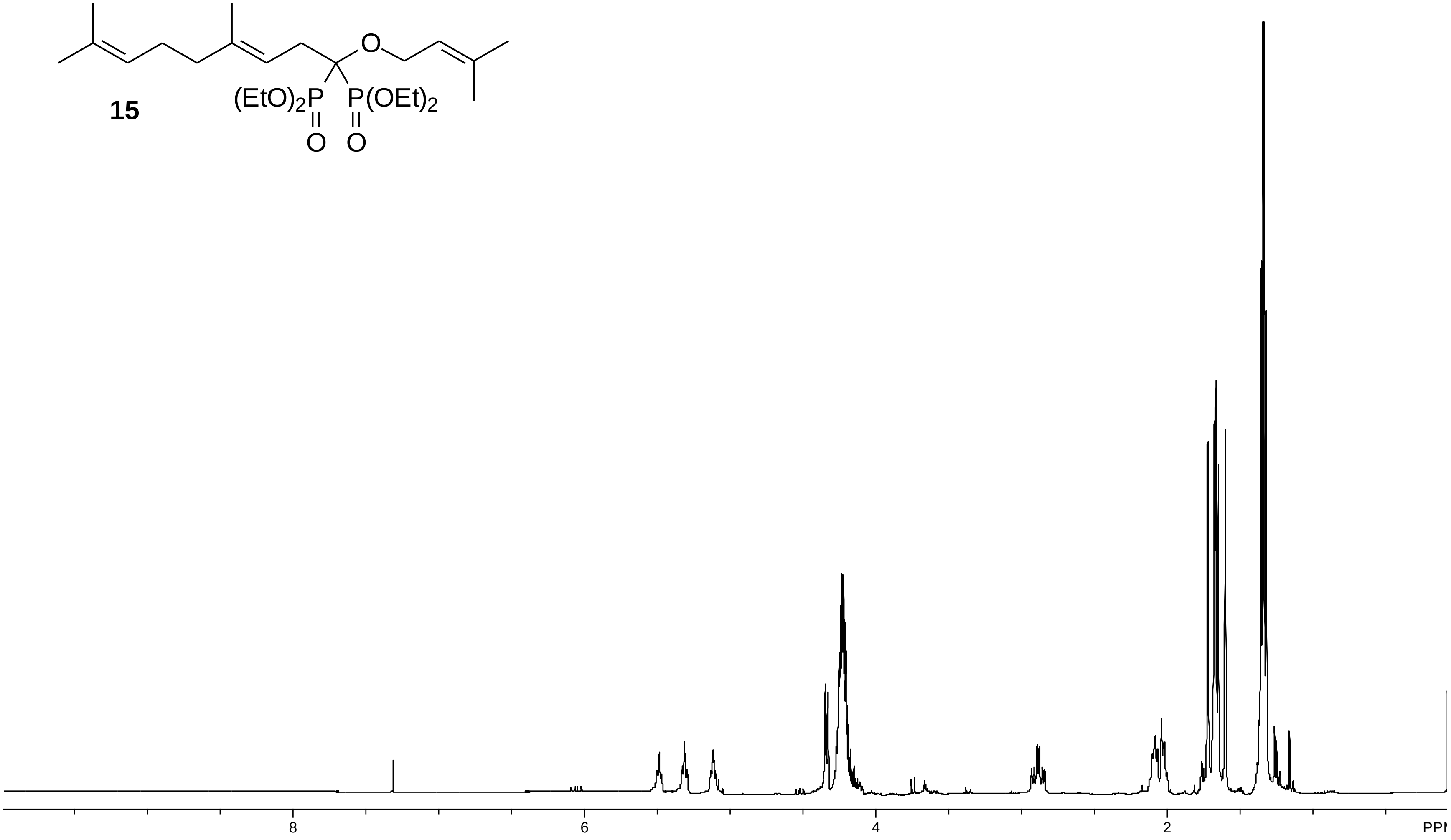

400 MHz ^{1}H NMR Spectrum of Compound **15**.

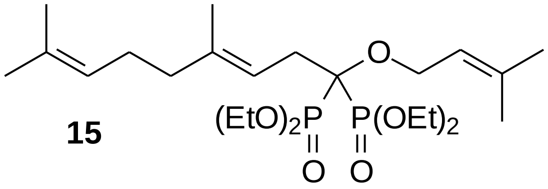

15

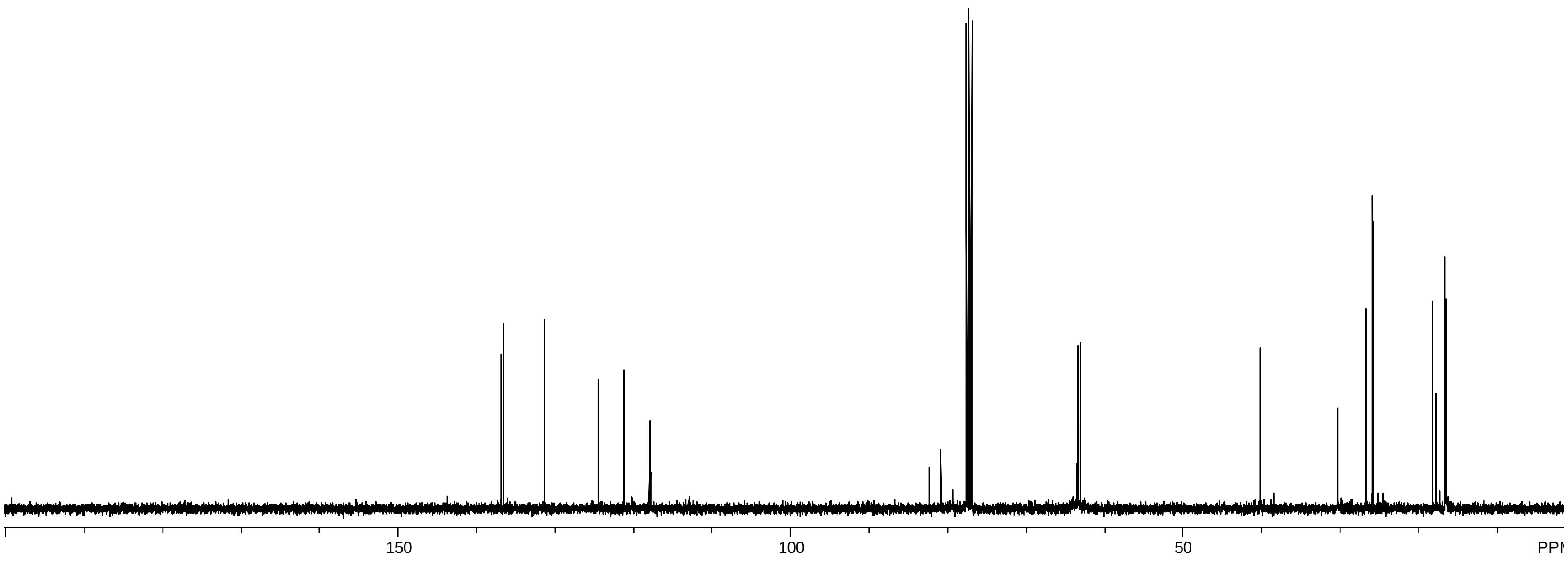

100 MHz ^{13}C NMR Spectrum of Compound **15**.

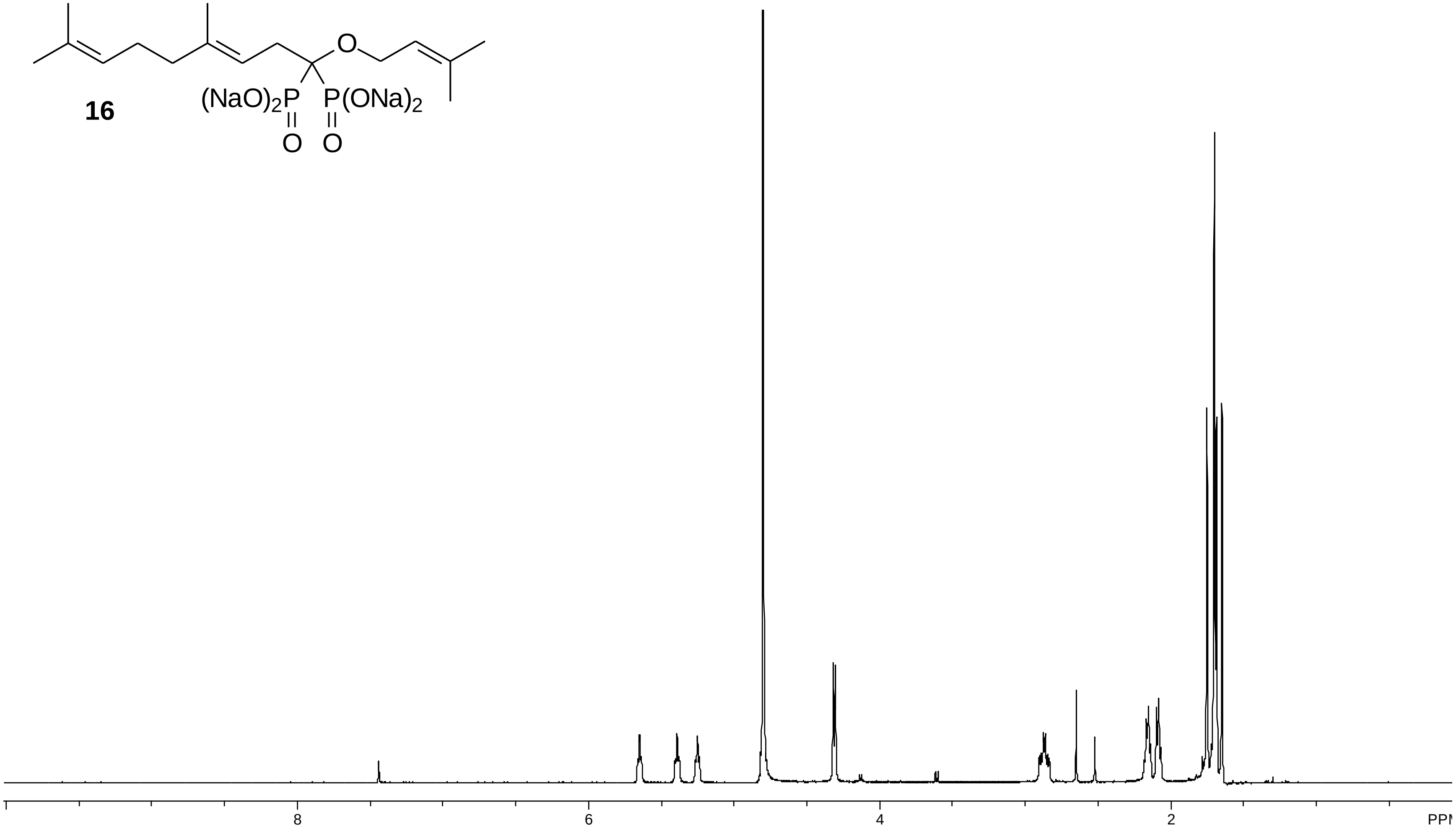

500 MHz ^{1}H NMR Spectrum of Compound **16**.

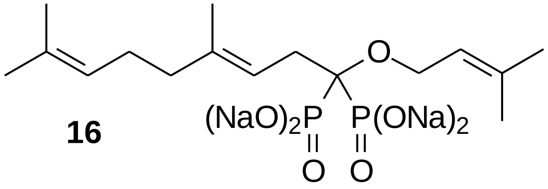

16

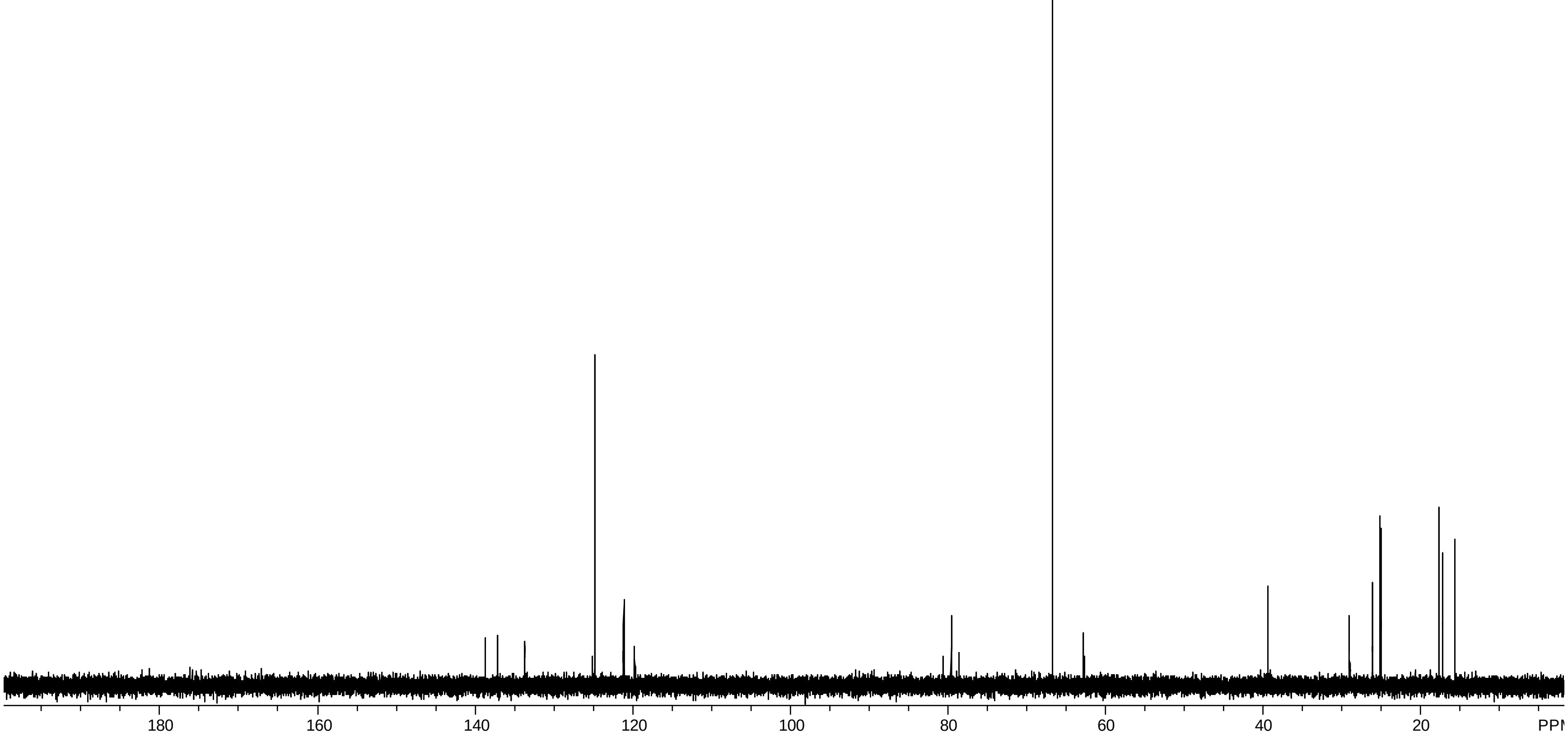

125 MHz ^{13}C NMR Spectrum of Compound **16**.

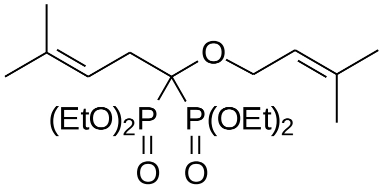

17

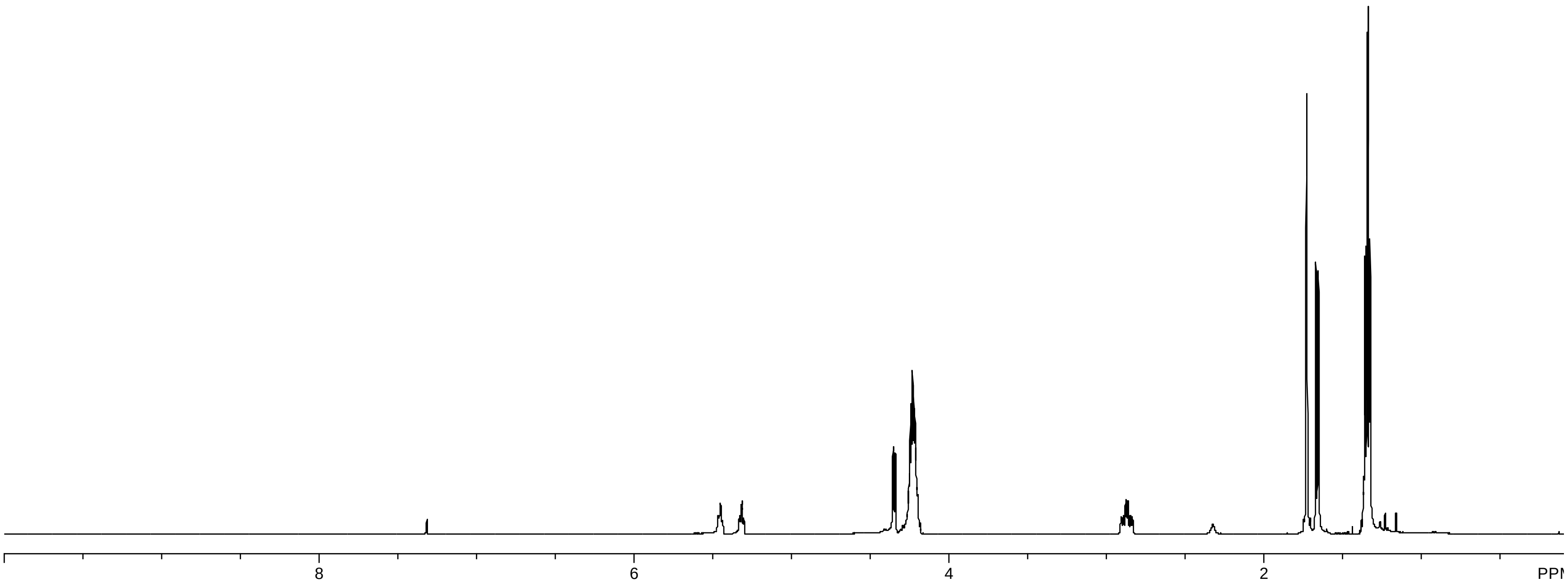

500 MHz ^{1}H NMR Spectrum of Compound **17**.

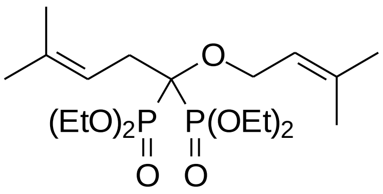

17

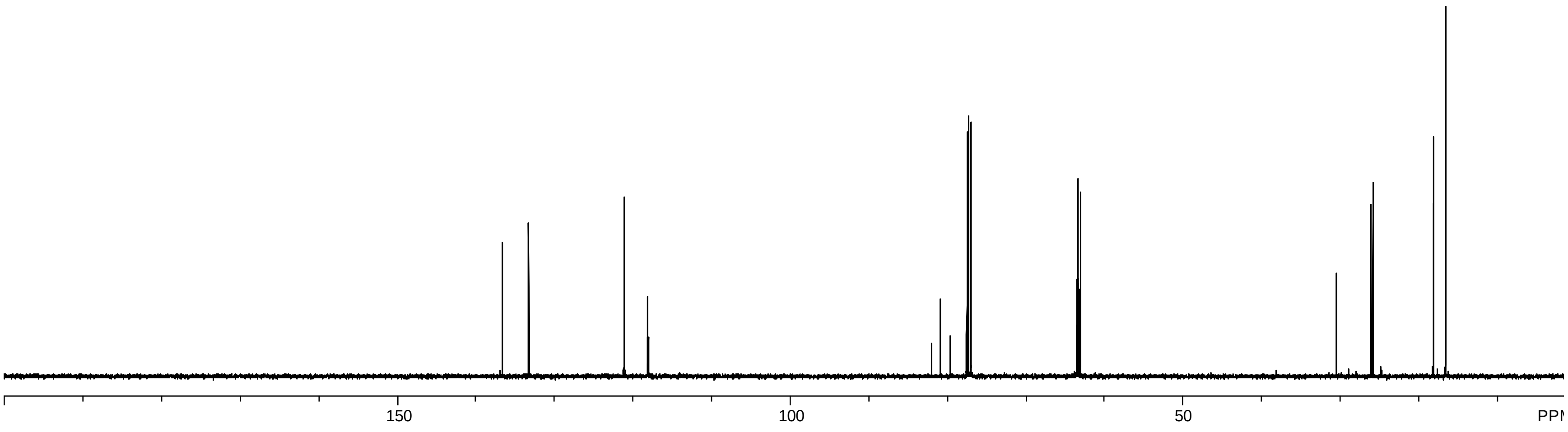

125 MHz ^{13}C NMR Spectrum of Compound **17**.

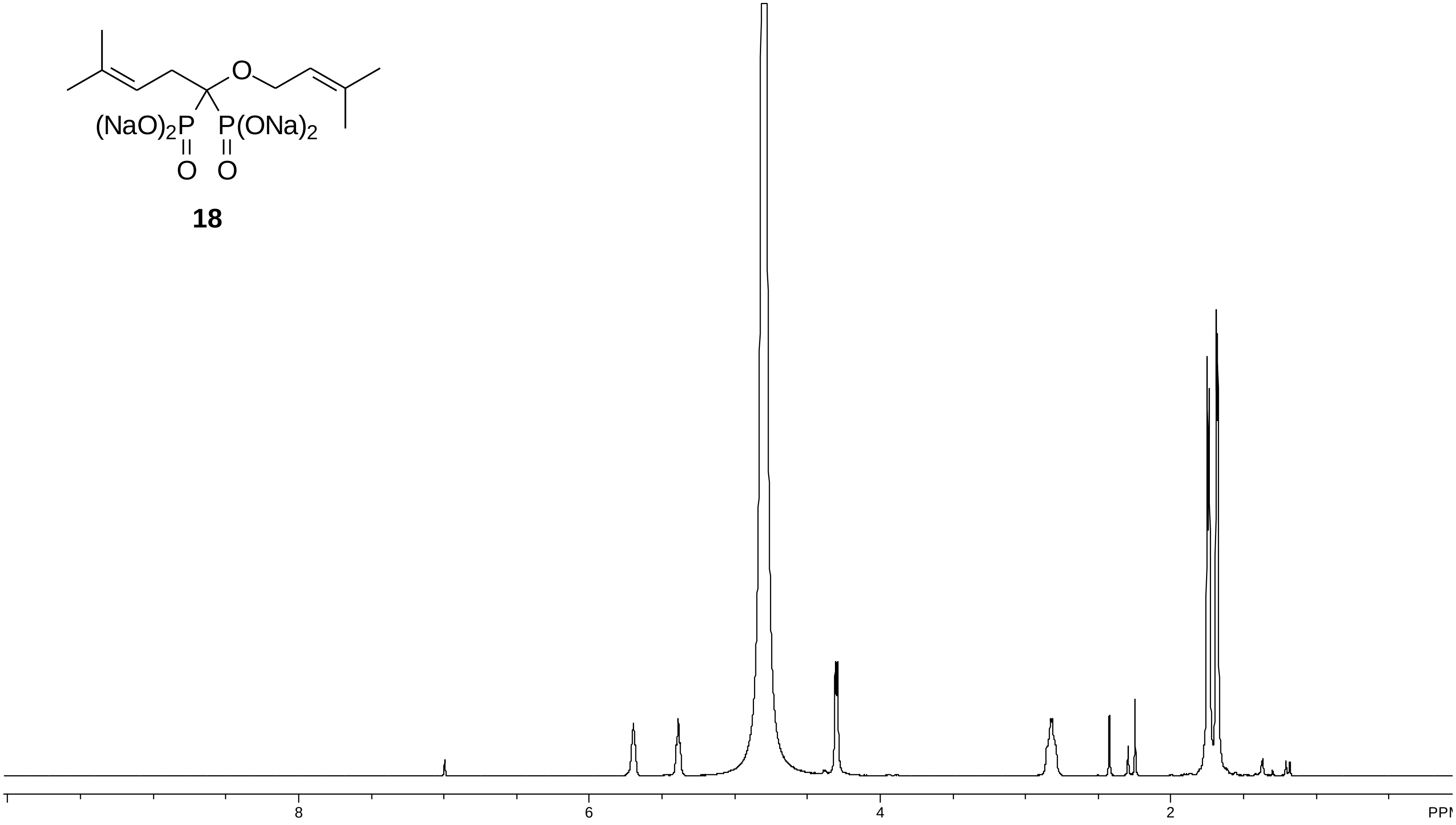

500 MHz ^{1}H NMR Spectrum of Compound **18**.

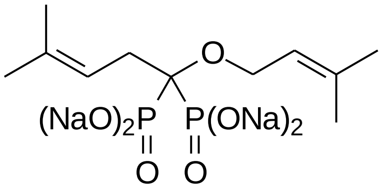

18

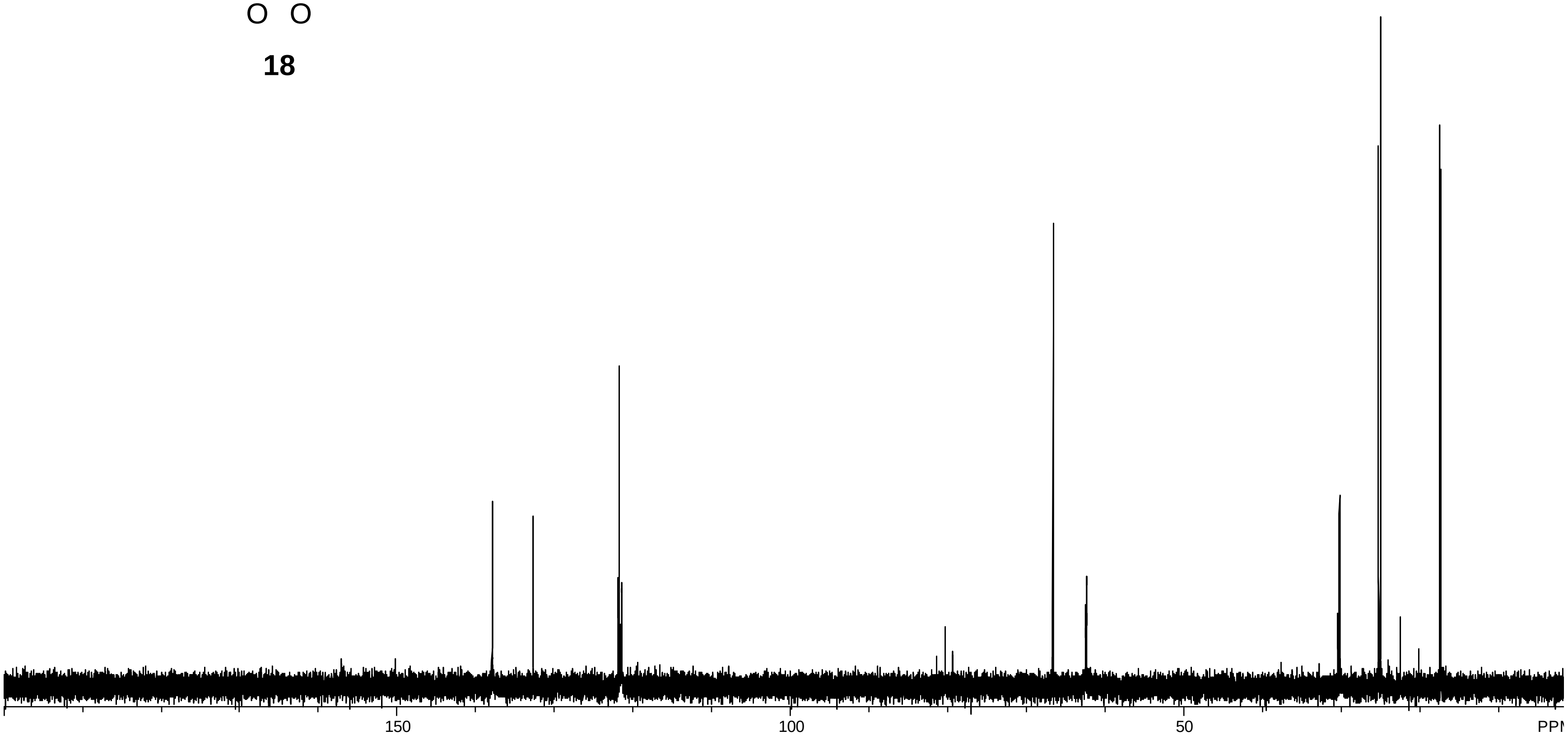

125 MHz ^{13}C NMR Spectrum of Compound **18**.

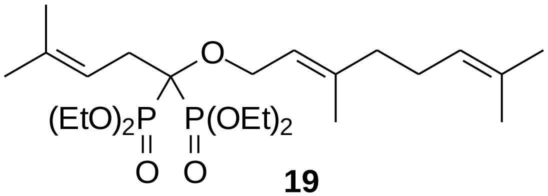

19

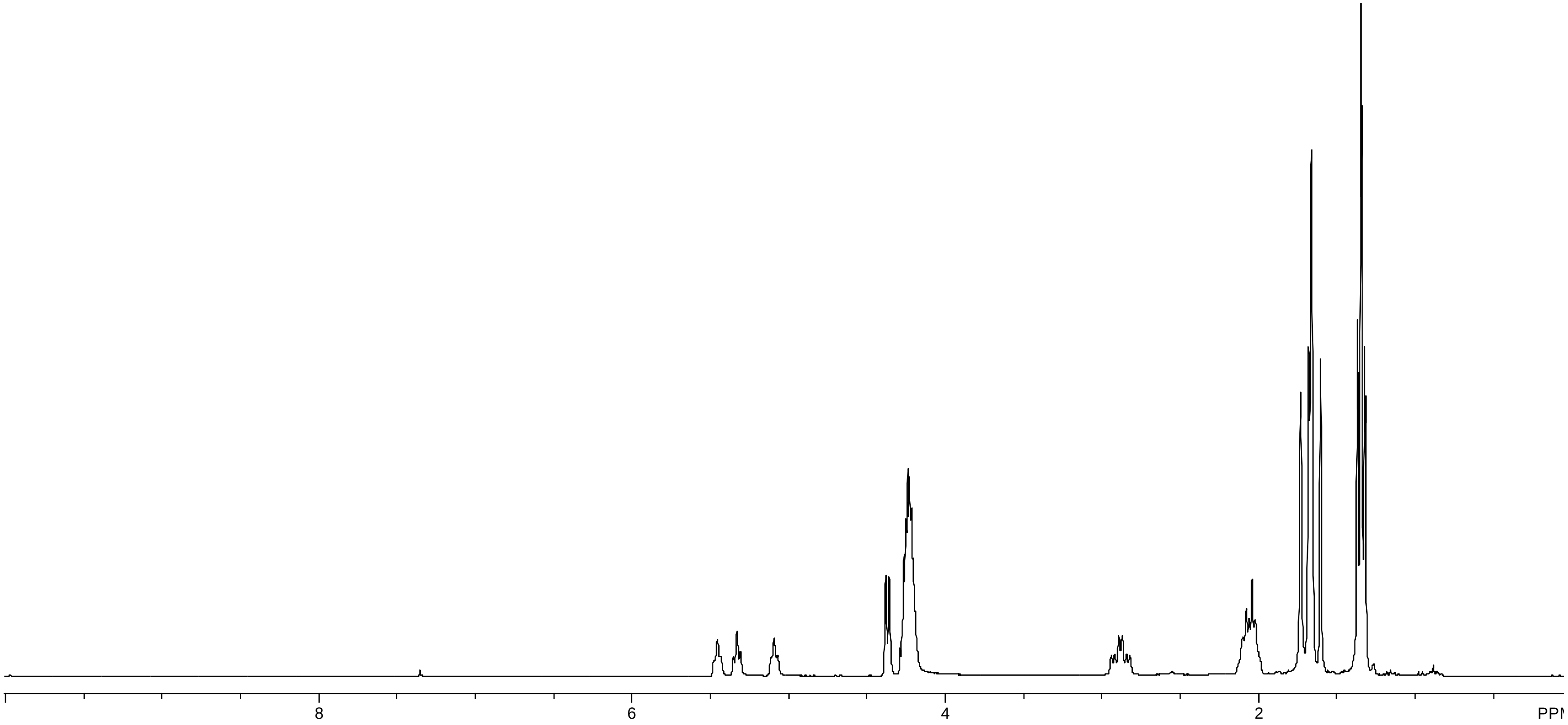

300 MHz ^{1}H NMR Spectrum of Compound **19**.

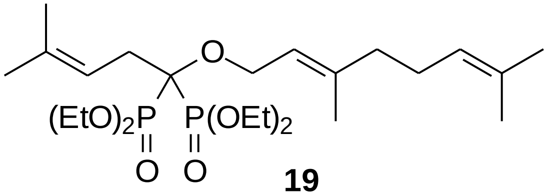

19

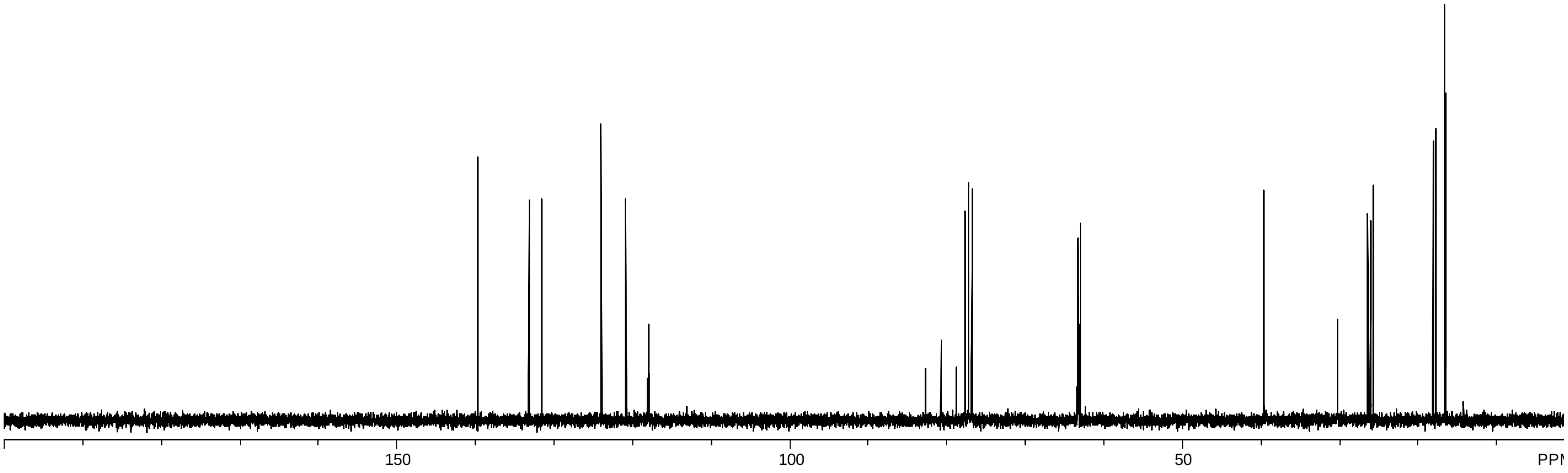

75 MHz ^{13}C NMR Spectrum of Compound **19**.

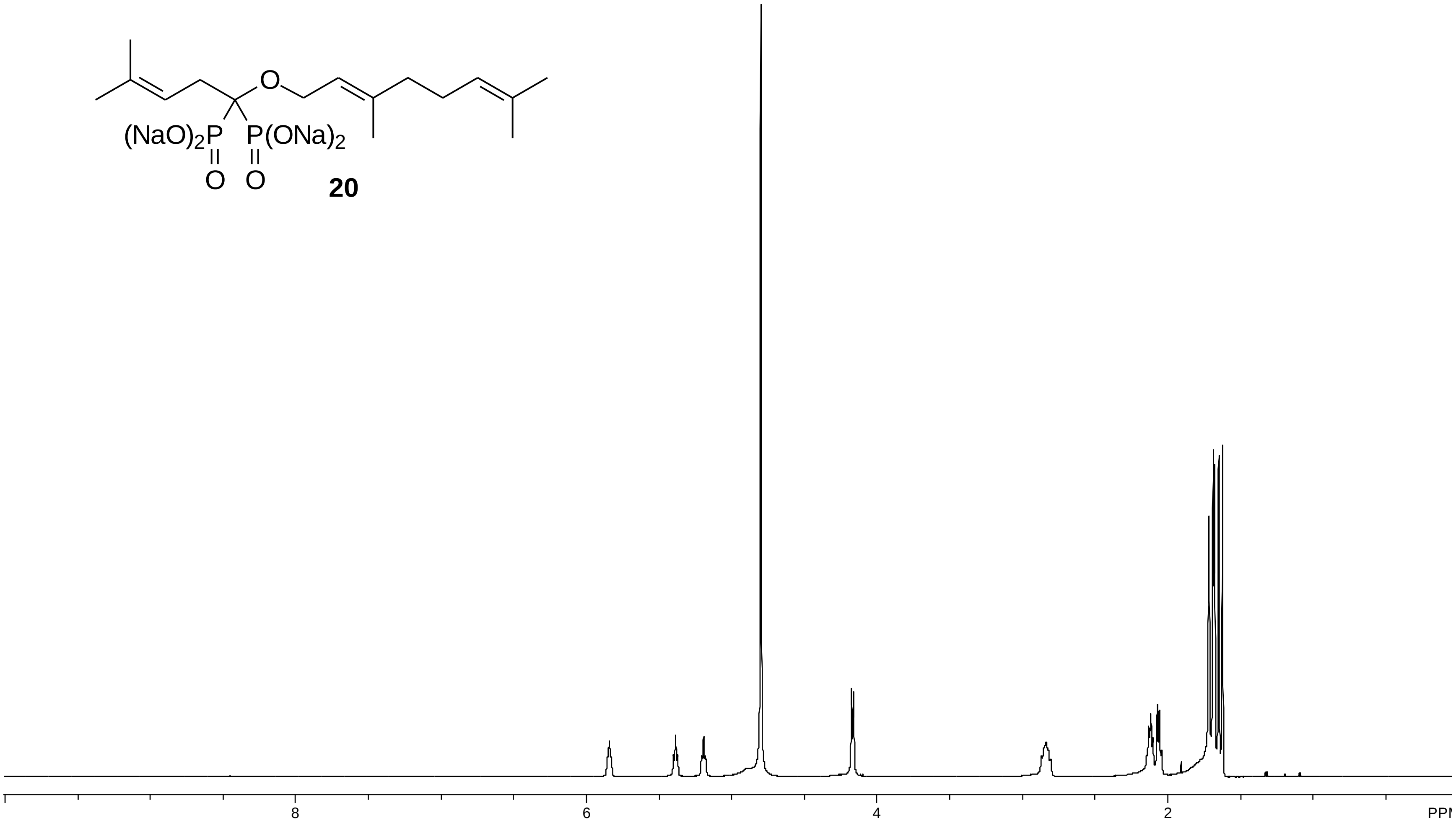

500 MHz ^{1}H NMR Spectrum of Compound **20**.

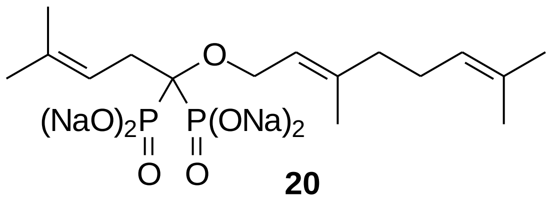

20

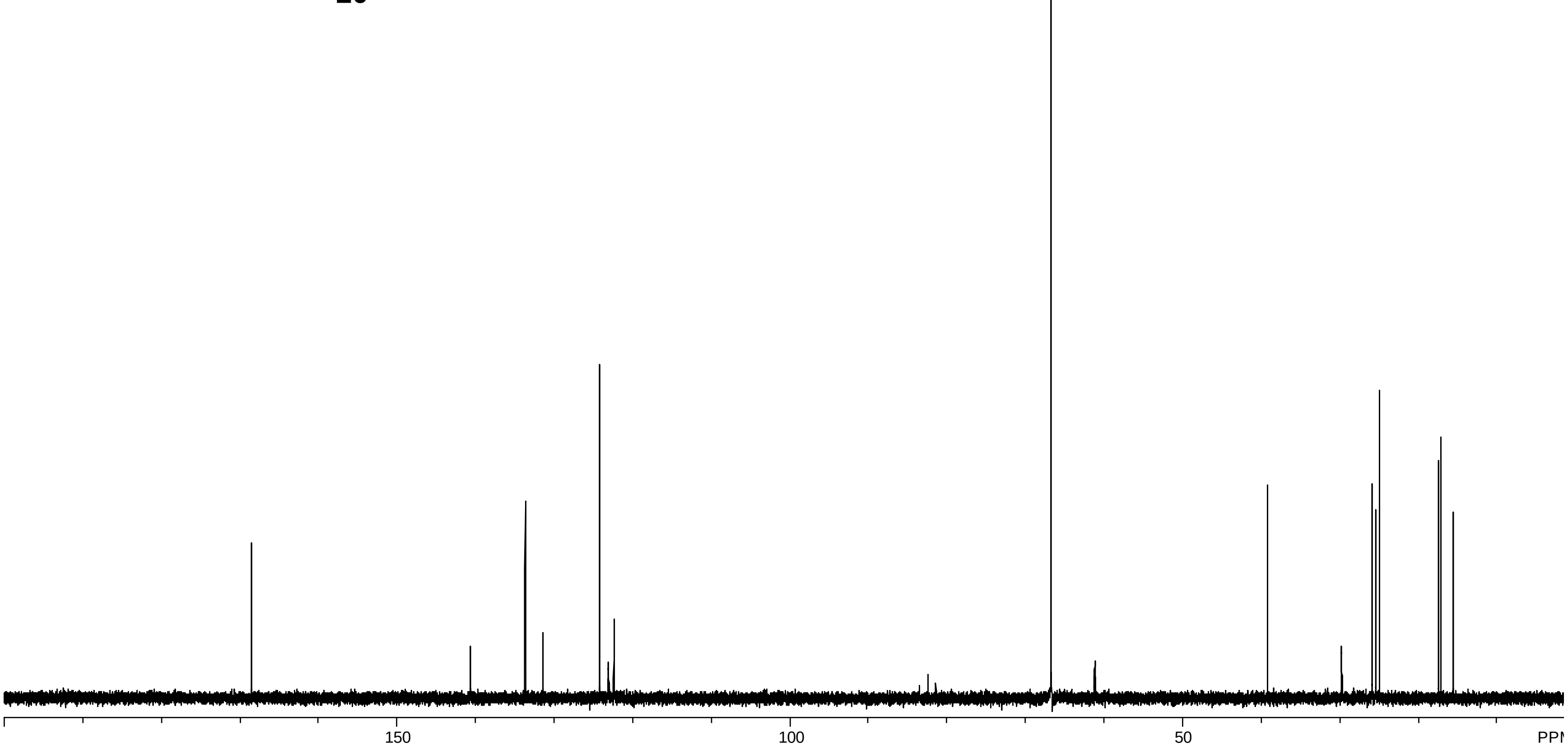

125 MHz ^{13}C NMR Spectrum of Compound **20**.

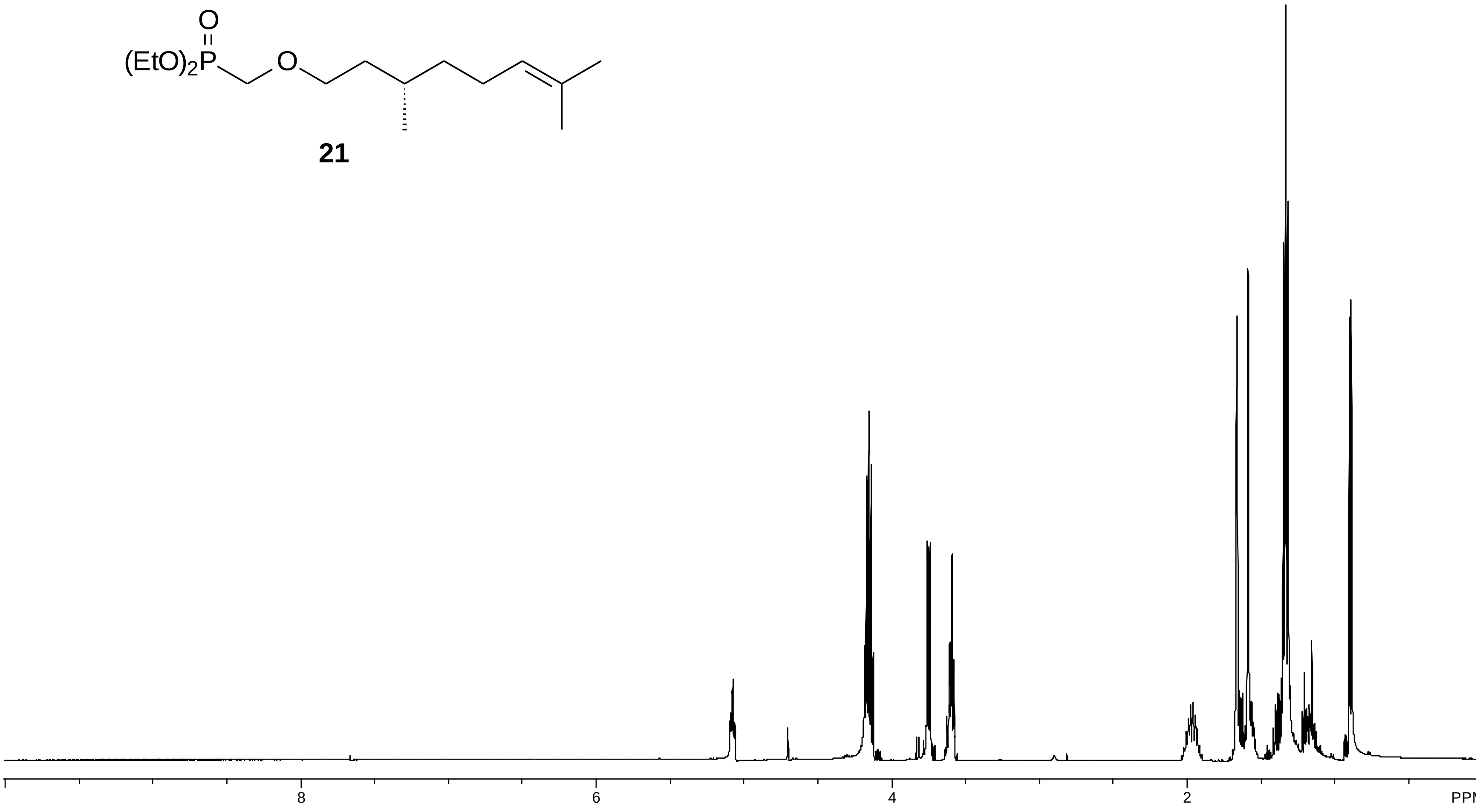

500 MHz ^{1}H NMR Spectrum of Compound **21**.

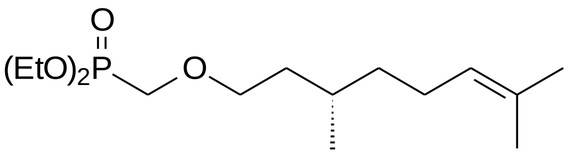

21

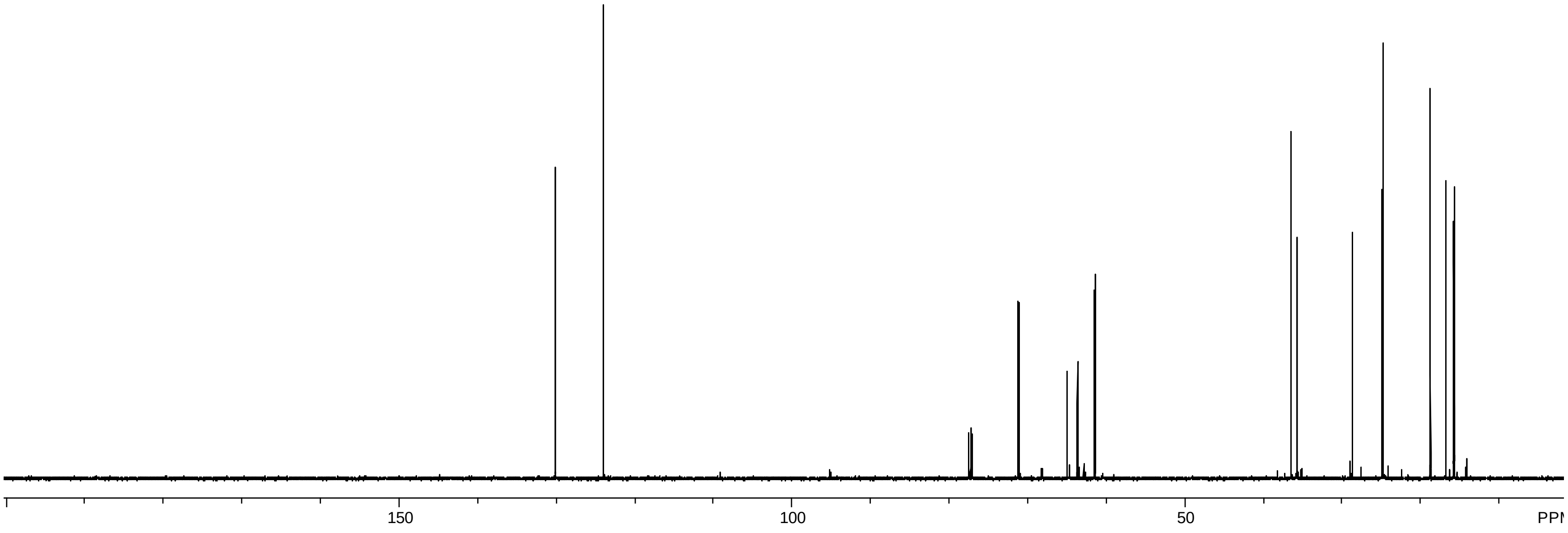

125 MHz ^{13}C NMR Spectrum of Compound **21**.

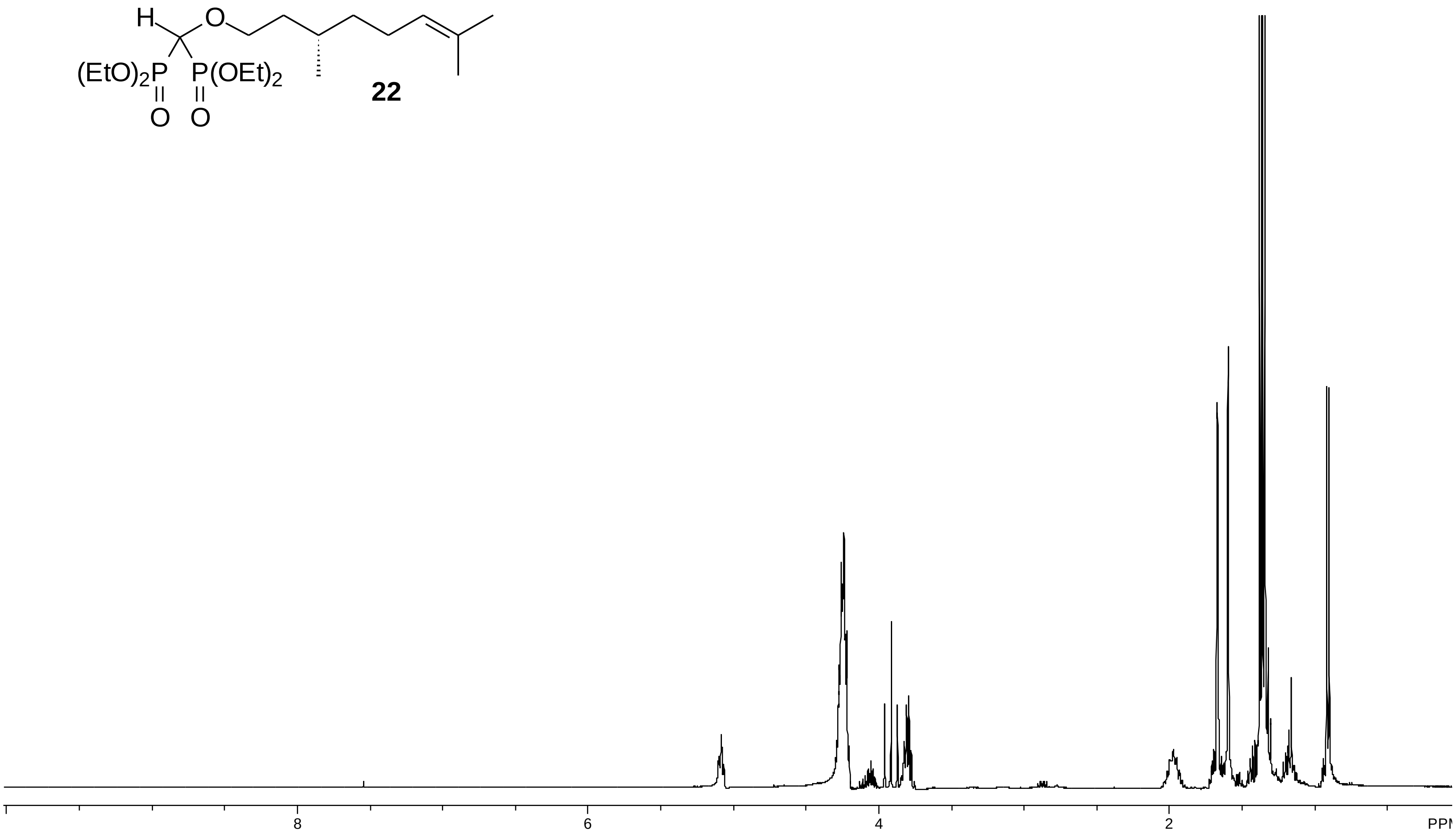

22

400 MHz ^{1}H NMR Spectrum of Compound **22**.

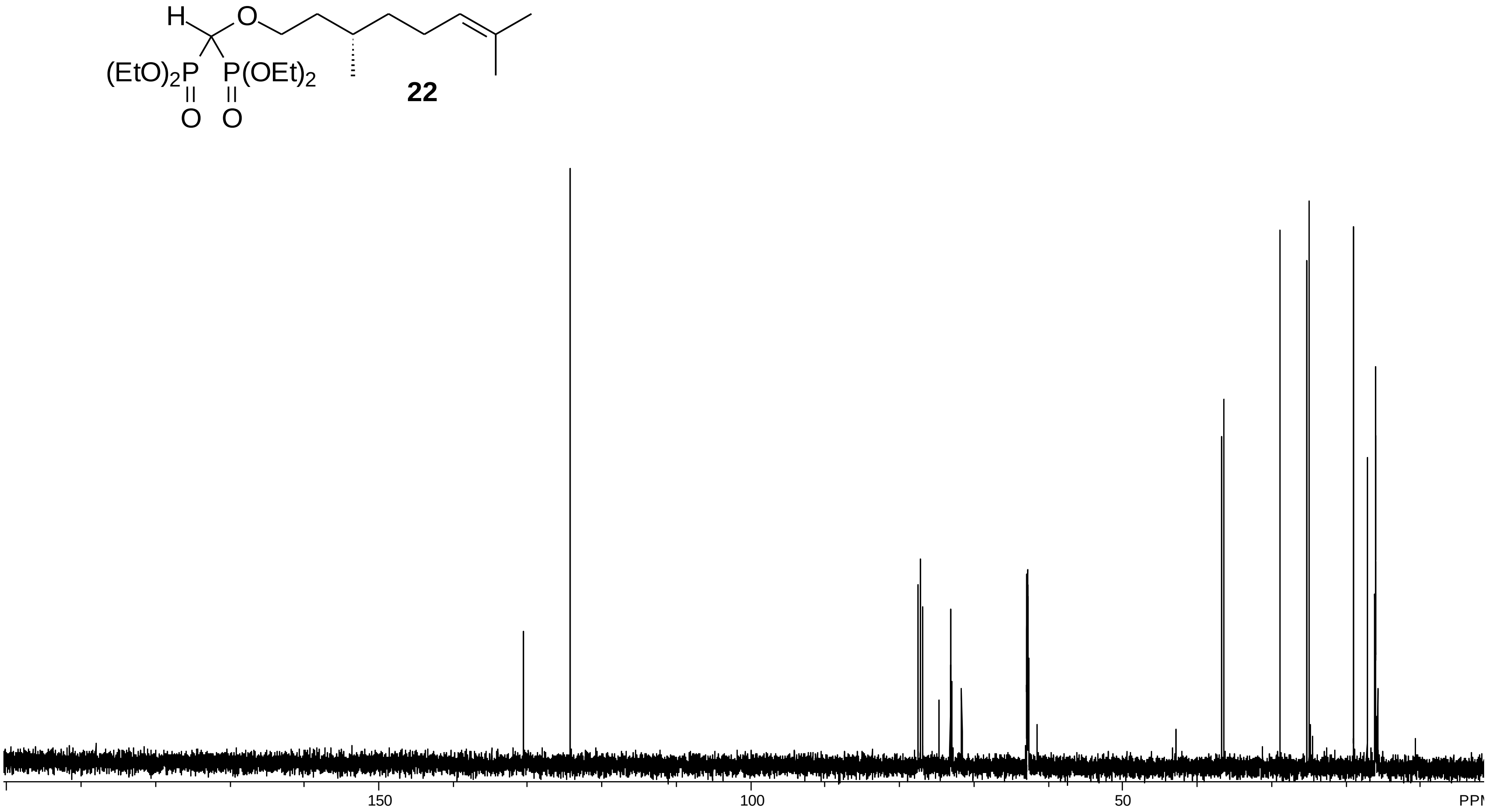

22

100 MHz ^{1}H NMR Spectrum of Compound **22**.

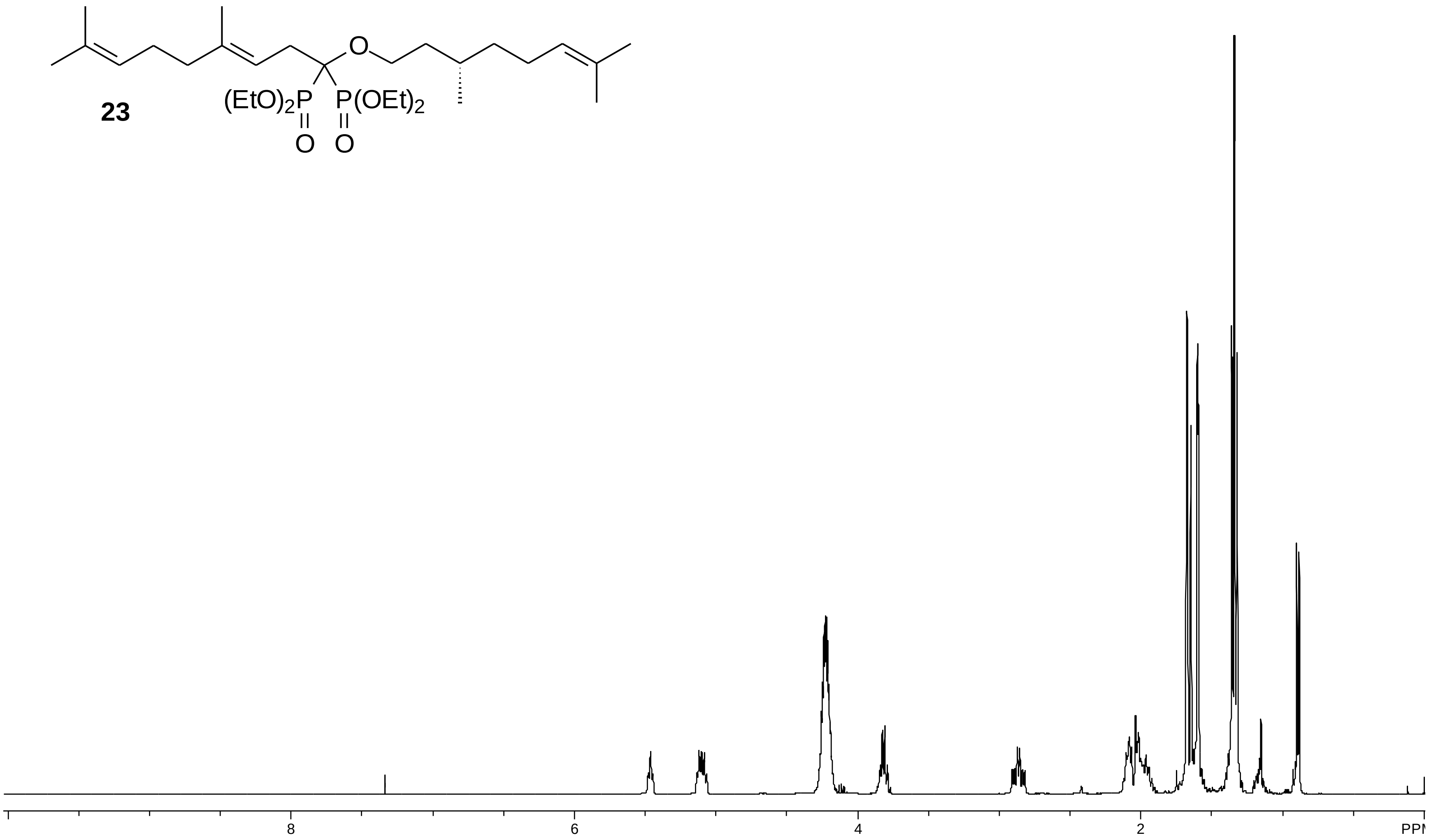

400 MHz ^{1}H NMR Spectrum of Compound **23**.

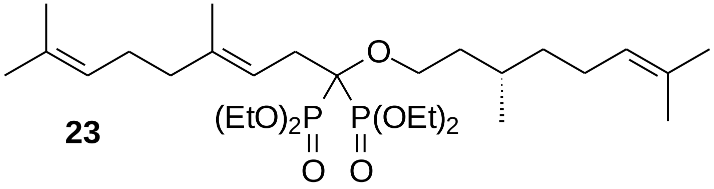

23

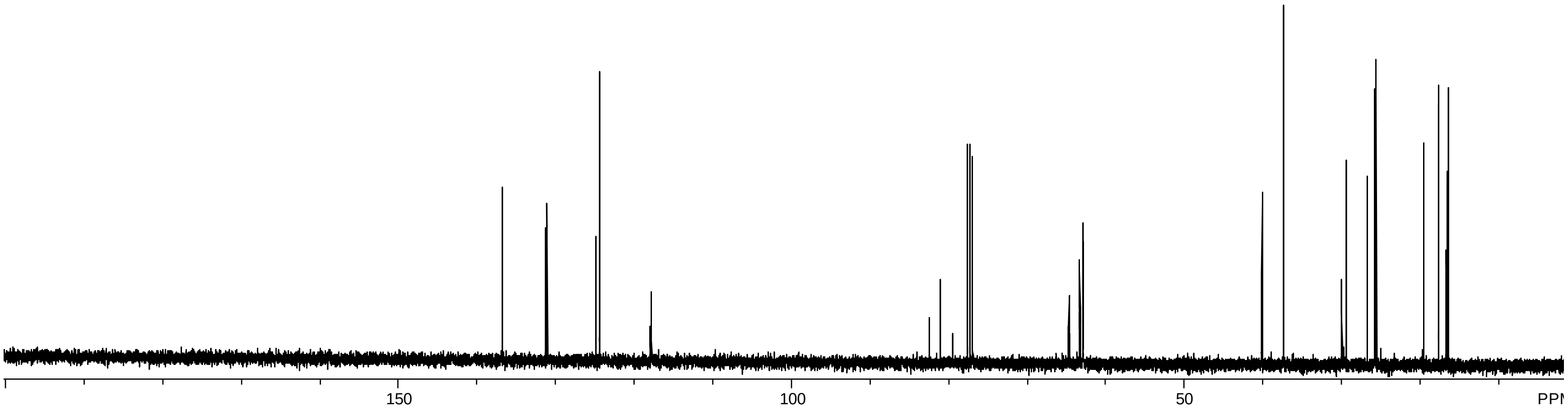

100 MHz ^{13}C NMR Spectrum of Compound **23**.

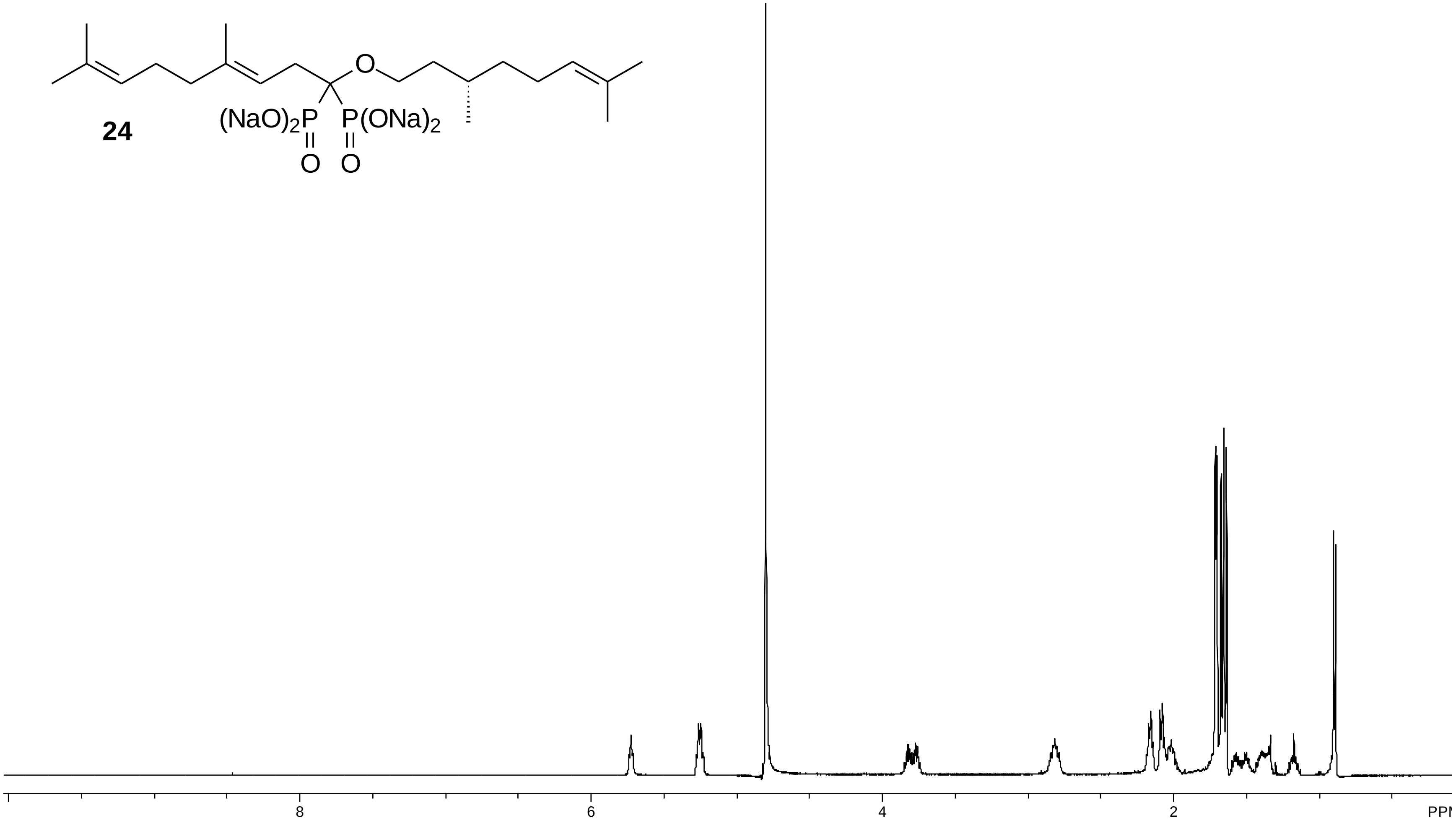

500 MHz ^{1}H NMR Spectrum of Compound **24**.

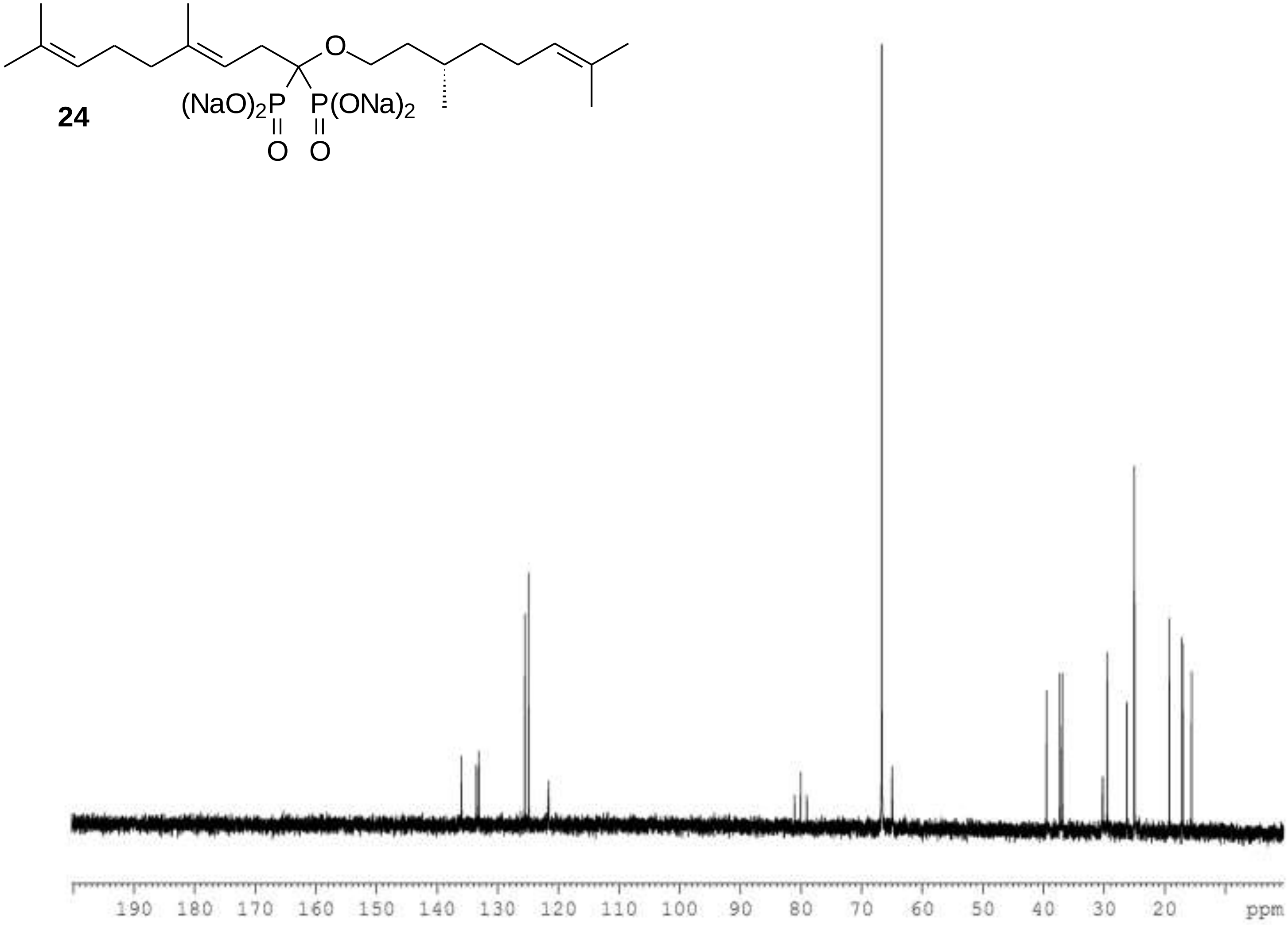

125 MHz ^{13}C NMR Spectrum of Compound **24**.
